# Effects of SGLT2 inhibitors on haematocrit and haemoglobin levels and the associated cardiorenal benefits in T2DM patients: A meta‐analysis

**DOI:** 10.1111/jcmm.17115

**Published:** 2021-12-08

**Authors:** Qi Tian, Keyu Guo, Jiayi Deng, Yanjun Zhong, Lin Yang

**Affiliations:** ^1^ National Clinical Research Center for Metabolic Diseases Key Laboratory of Diabetes Immunology, Ministry of Education Department of Metabolism and Endocrinology The Second Xiangya Hospital of Central South University Changsha Hunan China; ^2^ ICU Center The Second Xiangya Hospital of Central South University Changsha Hunan China

**Keywords:** cardiorenal protection, haematocrit, haemoglobin, meta‐analysis, SGLT2 inhibitors, type 2 diabetes mellitus

## Abstract

To explore the effect and magnitude of effect of sodium‐glucose cotransporter‐2 (SGLT2) inhibitors on haematocrit and haemoglobin and the related cardiorenal benefits in patients with type 2 diabetes mellitus (T2DM), PubMed, Web of Science, CENTRAL and EMBASE were searched to identify eligible trials. Weighted mean differences (WMDs) with 95% confidence intervals (CIs) were calculated using a random‐effects model. Seventy‐eight studies were included in the meta‐analysis. SGLT2 inhibitors significantly increased haematocrit and haemoglobin levels compared with control (total WMD 2.27% [95% CI 2.08, 2.47] and 6.20 g/L [95% CI 5.68, 6.73], respectively). Except for dapagliflozin (*p* = 0.000), no notable dose‐dependent relationship was revealed for other SGLT2 inhibitors. The effect could be sustained or even slightly increased with long‐term therapy (coef. =0.009, 95% CI [0.005, 0.013], *p* = 0.000). In subgroup analyses, haematocrit elevation increased with higher body mass index (BMI). A greater haematocrit elevation could be observed in white patients or when compared with active controls. In conclusion, SGLT2 inhibitors increased haematocrit and haemoglobin levels in T2DM patients. Changes in haematocrit and haemoglobin seem to be surrogate markers of improvement in renal metabolic stress, and important mediators involved in cardiorenal protection.

## 1 INTRODUCTION

Sodium‐glucose cotransporter‐2 (SGLT2) inhibitors exert glucose‐lowering effects by inhibiting SGLT2, which mediates glucose and sodium reuptake in the renal proximal tubule.^1^ Many large clinical trials have shown promising benefits of SGLT2 inhibitors on cardiovascular and renal outcomes in participants with type 2 diabetes mellitus (T2DM) in addition to its safe and effective glucose‐lowering effects.^2^, ^3^, ^4^, ^5^ The cardiorenal protective mechanisms of SGLT2 inhibitors have not been entirely clarified, and the possible mechanisms are multifactorial: improvements in various cardiovascular risk factors, such as hyperglycaemia, dyslipidaemia, hypertension, obesity, hyperuricaemia and albuminuria; reductions in oxidative stress, inflammation, apoptosis and mitochondrial dysfunction; correction of abnormal glomerular haemodynamics; and protection of cardiac structure and function by means of improving myocardial ischaemia, reducing ventricular load and improving myocardial metabolism.^6^, ^7^, ^8^, ^9^, ^10^, ^11^

Recently, a post hoc mediation analysis from the EMPA‐REG OUTCOME trial prompted the idea that changes in haematocrit and haemoglobin mediated approximately half of the decrease in the risk of cardiovascular mortality associated with empagliflozin.^12^ Coincidentally, markers of volume status and haematopoiesis exerted a strong mediating effect on heart failure and kidney protection, as revealed by two mediation analysis studies from the CANVAS Program.^13^, ^14^ Although there is sufficient evidence of associations between increased haematocrit and haemoglobin and SGLT2 inhibitor therapy amongst patients with T2DM, increases in haematocrit have not been consistently observed in several studies.^15^ In addition, data on the impact of such changes in haematological parameters on cardiorenal protection in T2DM patients are still lacking. Hence, we undertook a meta‐analysis to explore the effects of SGLT2 inhibitors on erythropoiesis parameters and the associated beneficial cardiorenal protection effects in T2DM patients.

## 2 METHODS

### 2.1 Search strategy

We carried out literature searches in PubMed, Web of Science, the Cochrane Central Register of Controlled Trials (CENTRAL) and EMBASE from database inception to March 8, 2021. MeSH terms and free‐text terms associated with each gliflozin were used. The complete search strategy is presented in Table S1. Moreover, we manually scanned references from retrieved trials, relevant meta‐analyses and reviews to search for additional reports.

### 2.2 Inclusion and exclusion criteria

Trials fulfilling the following inclusion criteria were included: (1) randomized controlled trials (RCTs) conducted with participants with T2DM comparing SGLT2 inhibitors, either as monotherapy or as an add‐on to other hypoglycaemic drugs or insulin, with placebo, active control or standard treatment; (2) mean (SD) changes from baseline in levels of erythropoiesis parameters reported for every group or other data allowing for the calculation of the above variables. The primary outcomes were mean (SD) changes in haematocrit and haemoglobin levels, and the secondary outcomes included mean (SD) changes in erythrocytes, reticulocytes and erythropoietin (EPO) levels from baseline. We excluded observational studies, pooled analyses, noncontrolled or nonrandomized trials, articles enrolling nondiabetic or patients with type 1 diabetes mellitus (T1DM) or articles not reporting the outcomes of interest.

### 2.3 Data extraction and quality assessment

Data were extracted from the full‐text and supplementary information of eligible publications according to a prespecified electronic data collection form. For each study, the following data were carefully extracted: first author, publication year, registration number, study design, sample size, baseline characteristics, types and dosages of SGLT2 inhibitors and control compound, treatment duration and changes (SD) in erythropoiesis parameters from baseline. The data from different study periods for the same subjects were presented together. The quality of RCTs was assessed by the Cochrane Collaboration Risk‐of‐Bias Tool consisting of five aspects: random sequence generation, allocation concealment, blinding, incomplete outcome data and selective reporting. Two authors (QT and KYG) independently extracted data and evaluated the quality of every RCT. If there were any divergences, an agreement was reached after discussion; otherwise, another author (LY) was consulted to resolve the conflict.

### 2.4 Statistical analysis

Data available at the first observation point were used, and a random‐effects model was applied to calculate weighted mean differences (WMDs) with 95% confidence intervals (CIs) between individual SGLT2 inhibitor and control groups. If SDs were not reported, we used relevant guidelines to calculate SDs.^16^ For trials providing median and range values, we estimated the mean and SD according to the appropriate formulas.^17^ If necessary, mean (SD) changes from baseline were imputed from baseline and endpoint values. Statistical heterogeneity was quantified using the I^2^ statistic (significant for I^2^ > 50%).^18^ Predefined subgroup analyses were carried out for various types and dosages of SGLT2 inhibitors. Sensitivity analysis was conducted utilizing the leave‐one‐out method. Meta‐regression analyses were conducted to assess any possible dose—and study period—dependency between each SGLT2 inhibitor and changes in the levels of haematopoietic parameters. To analyse the treatment duration, data from multiple intervention arms of one observation were collated to a single group, and different comparator data were also merged when needed. Additional subgroup analyses were employed to assess whether the effect size was related to the type of comparator or baseline characteristics. Moreover, studies with insulin as background therapy were analysed separately. Funnel plots and Egger's test were used to explore publication bias. All statistical analyses were performed with STATA 11.0 (Stata Corporation, TX, USA) and were reported according to the Preferred Reporting Items for Systematic Reviews and Meta‐analysis (PRISMA) guidelines. The PRISMA checklist for meta‐analysis is presented in Table S3. *P* < 0.05 indicated statistical significance.

## 3 RESULTS

### 3.1 Study selection and characteristics

**FIGURE 1:**
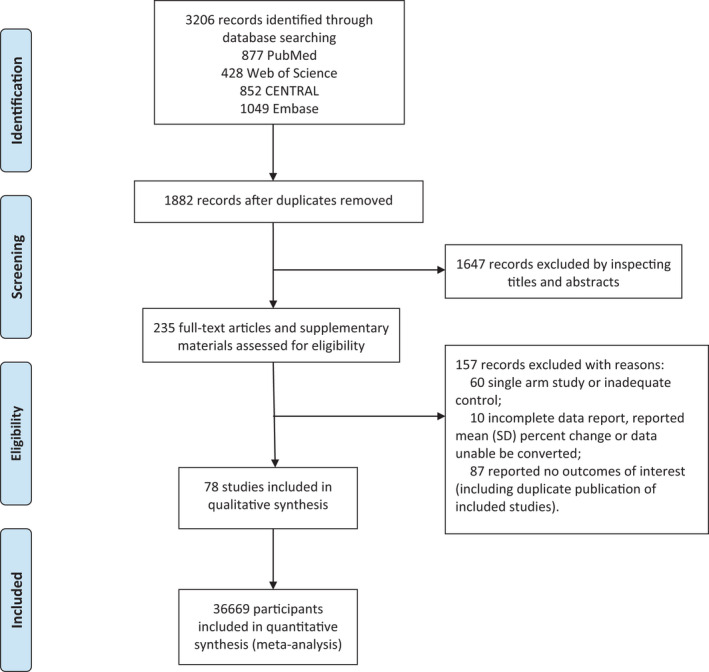
Flow diagram

**FIGURE 2:**
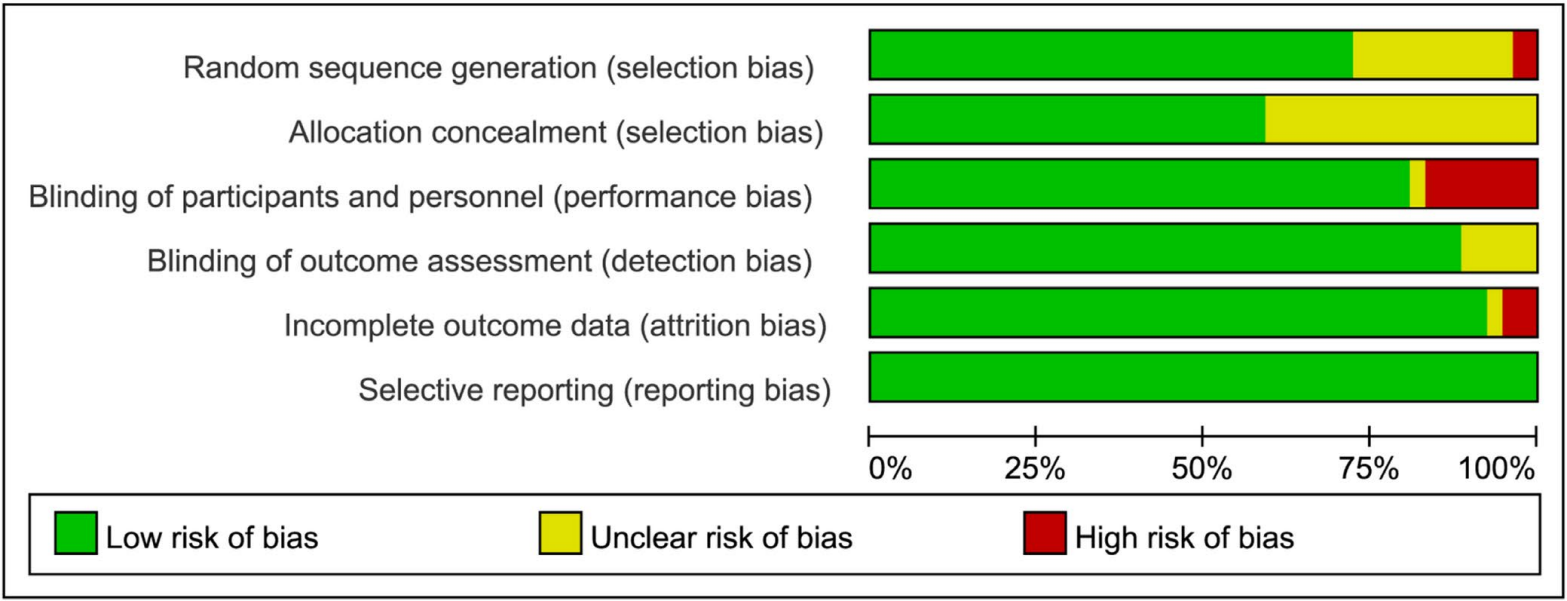
Risk‐of‐bias graph

Figure 1 shows the study selection process. Of 3206 records searched, 78 studies enrolling 36669 patients with T2DM were included in the meta‐analysis. No additional studies were identified by manual search. Table S2 exhibits the characteristics of the studies. Seven SGLT2 inhibitors (empagliflozin, dapagliflozin, canagliflozin, ipragliflozin, luseogliflozin, ertugliflozin and bexagliflozin) were studied. The duration of interventions varied from 4 to 208 weeks. The mean age, baseline glycated haemoglobin (HbA1c), body mass index (BMI) and estimated glomerular filtration ratio (eGFR) of the enrolled patients were 58.9 years, 8.1%, 30.1 kg/m^2^ and 78.6 ml/min/1.73 m^2^, respectively. The majority of trials included in the meta‐analysis were classified as having a low risk of bias. Except in 12 open‐label trials, subjects and staff in most trials were blinded. Details of bias assessments are presented in Figure 2.

### 3.2 Effects of SGLT2 inhibitors on haematocrit (%) and haemoglobin (g/L)

**FIGURE 3:**
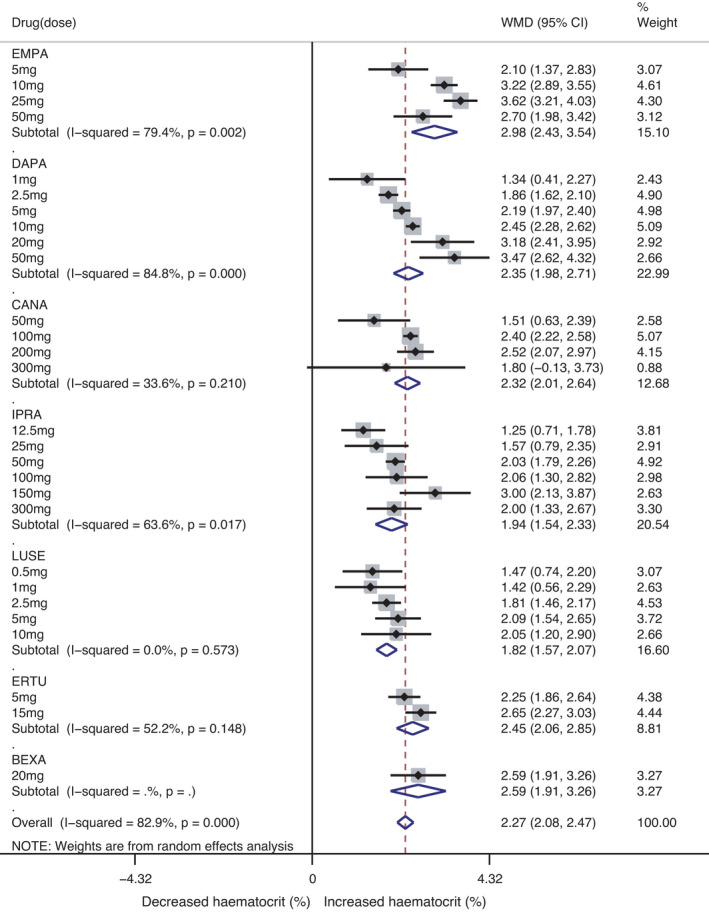
Meta‐analysis of WMD and 95% CI of changes in haematocrit (%) level for SGLT2 inhibitors, stratified by drug. The WMD of each dose was a combined result of multiple observations. WMDs are from a random‐effects model analysis. CI: confidence interval; WMD: weighted mean difference

Compared with the control, SGLT2 inhibitors markedly increased haematocrit levels (total WMD 2.27% [95% CI 2.08, 2.47], *p* = 0.000, I^2^ = 82.9%; empagliflozin WMD 2.98% [95% CI 2.43, 3.54], dapagliflozin WMD 2.35% [95% CI 1.98, 2.71], canagliflozin WMD 2.32% [95% CI 2.01, 2.64], ipragliflozin WMD 1.94% [95% CI 1.54, 2.33], luseogliflozin WMD 1.82% [95% CI 1.57, 2.07], ertugliflozin WMD 2.45% [95% CI 2.06, 2.85] and bexagliflozin WMD 2.59% [95% CI 1.91, 3.26]). The pooled WMD of the effect of SGLT2 inhibitors on haemoglobin was 6.20 g/L [95% CI 5.68, 6.73] (*p* = 0.000, I^2^ = 53.7%) across all studies and 7.21 g/L [95% CI 5.11, 9.30], 6.91 g/L [95% CI 5.90, 7.91], 6.95 g/L [95% CI 5.83, 8.07], 5.16 g/L [95% CI 4.04, 6.28], 5.58 g/L [95% CI 4.76, 6.41], 6.23 g/L [95% CI 5.30, 7.16] and 7.42 g/L [95% CI 4.87, 9.96] across studies using empagliflozin, dapagliflozin, canagliflozin, ipragliflozin, luseogliflozin, ertugliflozin and bexagliflozin, respectively. Effect sizes of certain doses are shown in Figure 3, S1.

### 3.3 Effects of SGLT2 inhibitors on erythrocytes (*10^12^/L), reticulocytes (*10^9^/L) and EPO (IU/L)

**FIGURE 4:**
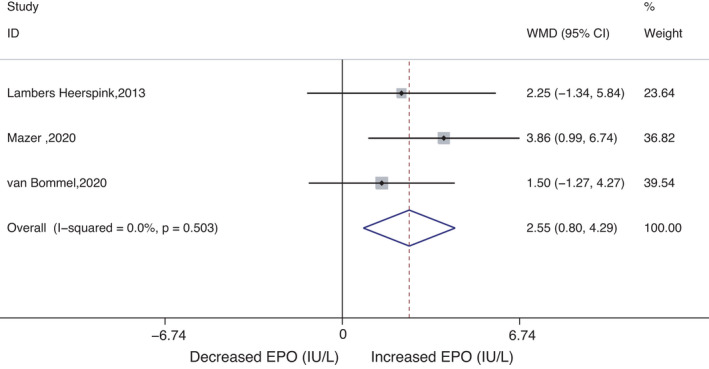
Meta‐analysis of WMD and 95% CI of changes in EPO (IU/L) levels for SGLT2 inhibitors. WMDs are from a random‐effects model analysis. CI: confidence interval; WMD: weighted mean difference

When evaluating the effects of SGLT2 inhibitors on erythrocytes, reticulocytes and EPO, different dosages of SGLT2 inhibitors were combined into one group. Twelve RCTs with 7357 patients revealed the effect of SGLT2 inhibitors on erythrocytes (total WMD 0.24 *10^12^/L [95% CI 0.21, 0.27], *p* = 0.000, I^2^ = 67.1%; Figure S2). In three studies including 1064 patients with T2DM, no statistically significant difference existed between the therapy and control groups in the change in reticulocyte levels (total WMD 3.91 × 10^9^/L [95% CI−1.39, 9.21], *p* = 0.149, I^2^ = 62.5%; Figure S3). Three RCTs provided information on changes in EPO (n = 216), which were significantly higher in the SGLT2 inhibitor group than in the control group (total WMD 2.55 IU/L [95% CI 0.80, 4.29], *p* = 0.004, I^2^ = 0.0%; Figure 4).

### 3.4 Subgroup analyses and sensitivity analysis

**FIGURE 5:**
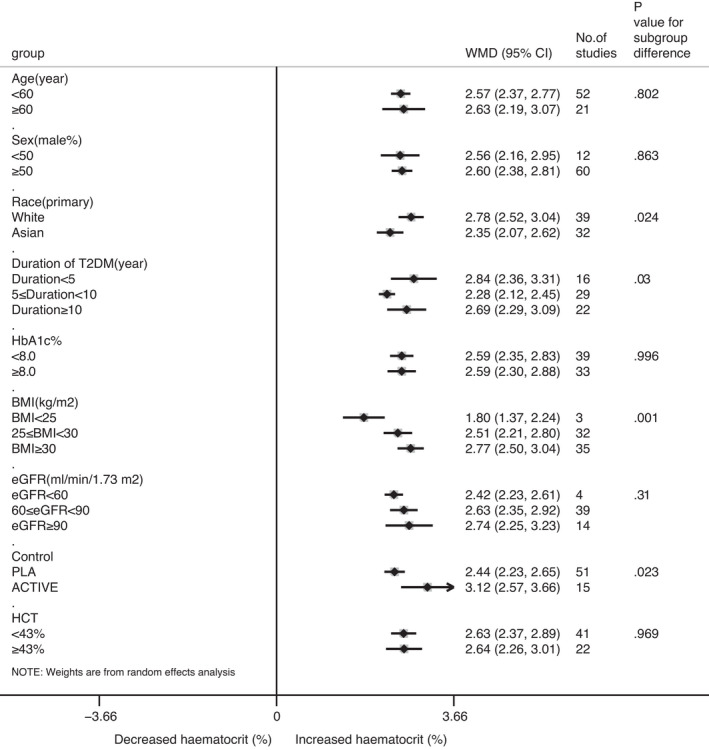
Subgroup analyses based on comparator type and baseline characteristics revealed that there was a significant difference in the increase in haematocrit levels between the treatment and control groups when stratified by race, type of the comparator, baseline duration of T2DM or BMI. No significant differences were found for age, sex, baseline HbAlc, eGFR or HCT. HbAlc: glycated haemoglobin; eGFR: estimated glomerular filtration rate; BMI: body mass index; HCT: haematocrit

Several prespecified subgroup analyses were performed to analyse heterogeneity of the effect of SGLT2 inhibitors on haematocrit. When stratified by race, comparator type, baseline duration of T2DM or BMI, there was a statistically significant difference in the increase in haematocrit levels between the therapy and control groups (Figure 5). No significant differences were found for age, sex, baseline HbA1c, eGFR or haematocrit. The effect of SGLT2 inhibitors combined with insulin therapy on haematocrit was also significant in seven RCTs (total WMD 2.55% [95% CI 1.82, 3.28] *p *= 0.000, I^2^ = 89.1%; Figure S4). In addition, we evaluated the robustness of the analysis results by means of a leave‐one‐out sensitivity analysis (data not presented).

### 3.5 Meta‐regression

A meta‐regression was conducted to assess whether the increase in haematocrit was dependent on the dose or duration of SGLT2 inhibitor therapy. Except for dapagliflozin, which exhibited a dose‐dependent relationship with haematocrit level (*p* = 0.000) (Figure [Link], [Link], [Link], [Link], [Link], [Link]), no notable relationship was found between the haematocrit‐increasing effect of individual SGLT2 inhibitors and the various doses (*p* > 0.05). Furthermore, irrespective of the type of SGLT2 inhibitor, meta‐regression was conducted to reveal the association between therapy effect and duration (coef. = 0.009, 95% CI [0.005, 0.013], *p* = 0.000; Figure S6), showing that the increase in the mean change in haematocrit could be sustained or even slightly increased with long‐term therapy.

### 3.6 Publication bias

No significant publication bias was observed. A symmetrical funnel plot revealed no potential publication bias for the comparison of haematocrit levels between the intervention and control groups, which was confirmed by Egger's test (*p* = 0.505) (Figure [Link], [Link]).

## 4 DISCUSSION

We demonstrated the effects of SGLT2 inhibitors on erythropoiesis parameters through a meta‐analysis including 78 RCTs. SGLT2 inhibitors significantly increased haematocrit and haemoglobin levels compared with the control condition (total WMD 2.27% [95% CI 2.08, 2.47] and 6.20 g/L [95% CI 5.68, 6.73], respectively). When stratified by drug type, we found a drug class effect on haematocrit and haemoglobin levels. The impacts on erythrocytes, reticulocytes and EPO in the form of total WMD were 0.24 × 10^12^/L [95% CI 0.21, 0.27], 3.91 × 10^9^/L [95% CI−1.39, 9.21] and 2.55 IU/L [95% CI 0.80, 4.29], respectively. The reason why the increase in reticulocyte count was not significantly different between the two groups might be related to the study duration. It is acknowledged that insulin therapy has a persistent antinatriuretic effect leading to elevated plasma volume.^19^, ^20^ Our meta‐analysis revealed that the effect of SGLT2 inhibitors combined with insulin on haematocrit was also significant in the subgroup analysis of seven RCTs (total WMD 2.55% [95% CI 1.82, 3.28]). In subgroup analyses, it was interesting that haematocrit elevation increased with higher BMI at baseline. So, to further investigate whether there was a lower haematocrit baselines in obese subjects, we correlated the mean of BMI and haematocrit baselines of each RCT and showed no statistically significant difference in the association between BMI and haematocrit (r = −0.224, *p* = 0.091). Owing to limited published data and only three RCTs with the mean of BMI baseline less than 25 kg/m^2^, we should be cautious about this result. Further studies are needed to investigate the phenomenon and clarify the underlying mechanisms.

Recently, a meta‐analysis on a similar topic was published, but the subjects studied were limited to T2DM patients with chronic kidney disease, and only four articles were included.^21^ Furthermore, the effect size calculated for the change in haemoglobin level was similar to the result of our study. However, analyses of the effects of SGLT2 inhibitors on erythrocytes, reticulocytes and EPO were absent. In the published meta‐analysis, most subjects were Caucasian; thus, the applicability of the study data to individuals of other ethnicities, such as Asians, was reduced. With the inclusion of only two SGLT2 inhibitors, empagliflozin and canagliflozin, no difference in the effects of different types of SGLT2 inhibitors on haematocrit and haemoglobin levels was observed.

Given that SGLT2 inhibitors induced glycosuria and natriuresis, increased haematocrit and haemoglobin levels were previously thought to result from haemoconcentration. However, this result may be only partly attributed to plasma volume contraction. First, the time course of changes in urine volume versus haematocrit (or haemoglobin) level differed substantially. Second, although diuretics had a more powerful diuretic effect, their application was not related to sustained elevation of haematocrit and haemoglobin levels.^22^, ^23^ Furthermore, there was evidence that increased levels of serum albumin, another plasma volume marker, were not consistent with those of haematocrit and haemoglobin.^24^ Finally, EPO, reticulocytes and haematocrit (or haemoglobin or red blood cells) are generally sequentially increased.^25^ Hence, the increase in haemoglobin and haematocrit depends on the enhancement of haematopoiesis, to a large extent. Although the change on EPO disappeared over 6–8 weeks,^22^ we could not rule out the sustained stimulatory effect of SGLT2 inhibitors on EPO secretion. With EPO decreasing, the haematocrit and haemoglobin levels maybe eventually return to baseline levels. However, previous studies showed the stable elevations in haematocrit and haemoglobin levels in patients receiving SGLT2 inhibitors over 104 weeks.^26^ The inhibition of hepcidin associated with enhanced haematopoiesis maybe contribute to this long‐term effect. Further work is needed to explore other mechanisms of this long‐term effect.

The mechanisms by which SGLT2 inhibitors augment erythropoiesis remain unclear. EPO is an integral erythropoietic hormone mainly arising from renal erythropoietin ‐producing cells (REPs). Hypoxia‐inducible factor (HIF) regulates EPO synthesis and secretion in a hypoxia‐inducible manner.^27^ EPO levels are usually low in diabetic patients, even in the presence of normal renal function.^28^ Proinflammatory molecules such as TNF‐α, IL‐1 and IL‐6, which are produced by stressed renal tubular epithelial cells, stimulate this conversion of REPs from hypoxia‐responsive cells to fibrogenic myofibroblasts that have decreased sensitivity to hypoxic response and produce inflammatory cytokines and fibrotic molecules instead of EPO.^29^, ^30^ In addition, inflammatory and fibrotic signals inhibit HIF through overactivation of prolyl hydroxylase domain (PHD) enzymes, even in a pathological hypoxic environment, which further impairs EPO synthesis and secretion capacity.^31^ It is gratifying that this shift from REPs into myofibroblasts is reversible and could potentially be reversed by SGLT2 inhibitors. T2DM often coexists with Non‐alcoholic fatty liver disease (NAFLD).^32^ Chronic low‐grade inflammation can affect the iron homeostasis by affecting the expression of hepcidin. Erythroferrone, a hormone that acts on hepatocytes to suppress the production of hepcidin, is regulated by EPO.^33^ So SGLT2 inhibitors induce an early increase in EPO, then elevate erythroferrone, and eventually suppress hepcidin. Besides, dapagliflozin inhibits de novo lipogenesis and increases fatty acid β‐oxidation by inducing glycosuria and increasing glucagon secretion from α‐cells in the pancreas, which attenuates liver steatosis and inflammation.^34^ Control of chronic inflammation related to fatty liver can also play a role in hepcidin downregulation. In sum, SGLT2 inhibitors may reduce glucose reabsorption in the proximal tubules, improve renal cortical hypoxia, diminish glucotoxicity, alleviate metabolic stress in renal proximal tubules and adjacent interstitium, promote the recovery of myofibroblasts to REPs, restore HIF activity, upregulate EPO synthesis and secretion, increase the absorption and utilization of iron by inhibiting the production of hepcidin and regulating other iron‐regulated proteins,^35^ and finally augment haematopoiesis.^36^, ^37^

The increase in haematopoietic parameters might have significant clinical implications, as shown in three post‐ hoc mediation analyses from the EMPA‐REG OUTCOME trial and CANVAS Program. Changes in haematocrit and haemoglobin appeared to be important mediators of the cardiorenal protective effects of SGLT2 inhibitors in univariate and multivariate models.^12^, ^13^, ^14^ The mechanism of the association between haematocrit and haemoglobin elevation and beneficial cardiorenal effects is less clear. Haematocrit and haemoglobin changes can be used as surrogate markers of alleviation of renal metabolic stress accompanied by treatment with SGLT2 inhibitors. The product of the haemoglobin value multiplied by the EPO value in the peripheral blood of clinical diabetic kidney disease (DKD) patients exhibits a good correlation with DKD stage and can predict the chance of chronic kidney failure in the future.^29^ Many studies have revealed that SGLT2 inhibitors inhibit the development and progression of chronic kidney disease.^38^, ^39^, ^40^ Moreover, renal cortical hypoxia in diabetic patients increases renal afferent sensory nerve activity, upregulating systemic sympathetic nervous system activity by communicating with the central nervous system. Hyperactivity of the sympathetic nervous system can be lowered by SGLT2 inhibitors, which partially explains the cardiovascular benefit. Accordingly, the kidney also plays a significant role in cardiovascular protection.^41^ Furthermore, elevated haemoglobin is beneficial for the improvement in tissue oxygenation in a damaged cellular environment, thereby exerting cardiorenal protection effects to some extent.

Several limitations of the meta‐analysis should be noted. First, significant heterogeneity existed and was not well explained by heterogeneity analyses. Second, almost none of the included trials were designed to assess the effects of SGLT2 inhibitors on increases in erythropoiesis parameters, and a majority of the analysed trials did not clarify the methodology used for the detection of erythropoiesis parameters. In addition, except for individual dose‐related studies, only 1 to 2 doses were generally analysed in the included studies, which may prevent the analysis of dose dependence. Furthermore, owing to limited published data, subgroup analyses were restricted to the mean of baseline characteristics rather than data obtained after grouping by baseline characteristics. Last, the changes observed in haematocrit and haemoglobin values were, in general, within the physiological range. The proportion of posttreatment polycythaemia was unknown, and it remained uncertain whether there was a significant difference in the magnitude of haematocrit and haemoglobin elevation at baseline in the presence or absence of anaemia.

In conclusion, SGLT2 inhibitors increase haematocrit and haemoglobin levels in patients with T2DM. With the exception of plasma volume contraction caused by diuresis, increased synthesis and secretion of EPO resulting from the improvement in metabolic stress following therapy with SGLT2 inhibitors plays a more important role. Changes in haematocrit and haemoglobin seem to be surrogate markers of improvement in renal metabolic stress and are associated with recovery from sympathetic hyperactivity. Thus, changes in haematocrit and haemoglobin are likely to be important mediators involved in cardiorenal protection. Specific mechanistic elucidation requires further research.

## CONFLICT OF INTEREST

All authors declared that there were no potential conflicts of interest.

## AUTHOR CONTRIBUTIONS

**Qi Tian:** Conceptualization (equal); Data curation (equal); Formal analysis (equal); Writing – original draft (lead). **Keyu Guo:** Data curation (equal); Formal analysis (equal). **Jiayi Deng:** Data curation (equal); Formal analysis (equal). **Yanjun Zhong:** Methodology (lead); Writing – review & editing (equal). **Lin Yang:** Conceptualization (equal); Supervision (lead); Writing – review & editing (equal).

## Supporting information

Fig S1

Fig S2

Fig S3

Fig S4

Fig S5

Fig S5

Fig S5

Fig S5

Fig S5

Fig S5

Fig S5

Fig S6

Fig S7a

Fig S7b

Fig S1‐S7

Table S1

Table S2

Table S3

## Data Availability

Data extracted or analysed in our work are included in the main text and supplementary files.
