# Supplementary figures and images for: Effects of SGLT2 inhibitors on haematocrit and haemoglobin levels and the associated cardiorenal benefits in T2DM patients: A meta‐analysis
Source: J Cell Mol Med. 2021 Dec 8;26(2):540–7. doi: 10.1111/jcmm.17115 (PMC8814934; doi:10.1111/jcmm.17115)

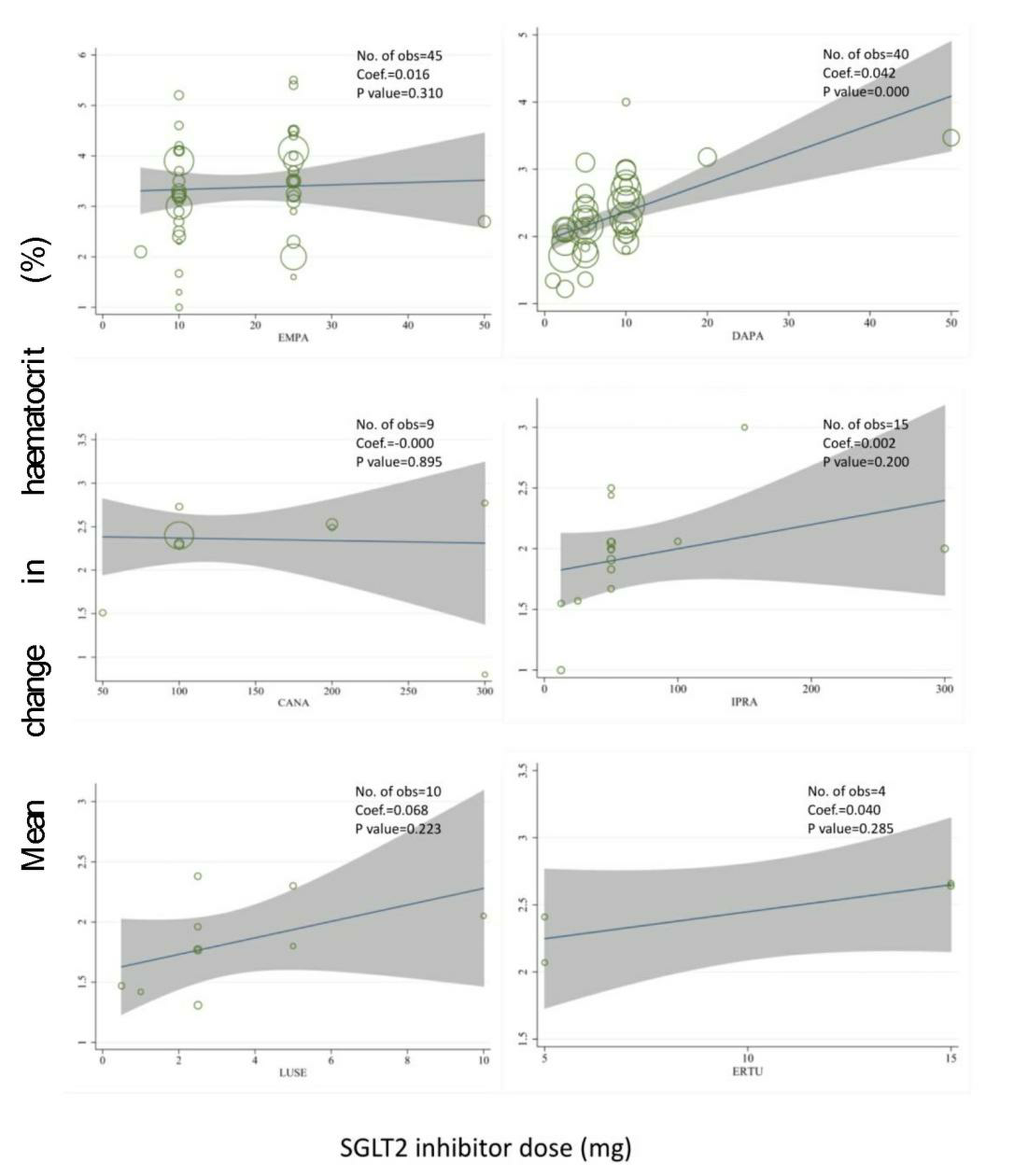

Supplement: Supplementary file 11 — Fig S5 [file JCMM-26-540-s012.png]
