# Supplementary material for: Effects of SGLT2 inhibitors on haematocrit and haemoglobin levels and the associated cardiorenal benefits in T2DM patients: A meta‐analysis
Source: J Cell Mol Med. 2021 Dec 8;26(2):540–7. doi: 10.1111/jcmm.17115 (PMC8814934; doi:10.1111/jcmm.17115)
Supplement: Supplementary file 15 — Fig S1‐S7 [file JCMM-26-540-s014.docx]

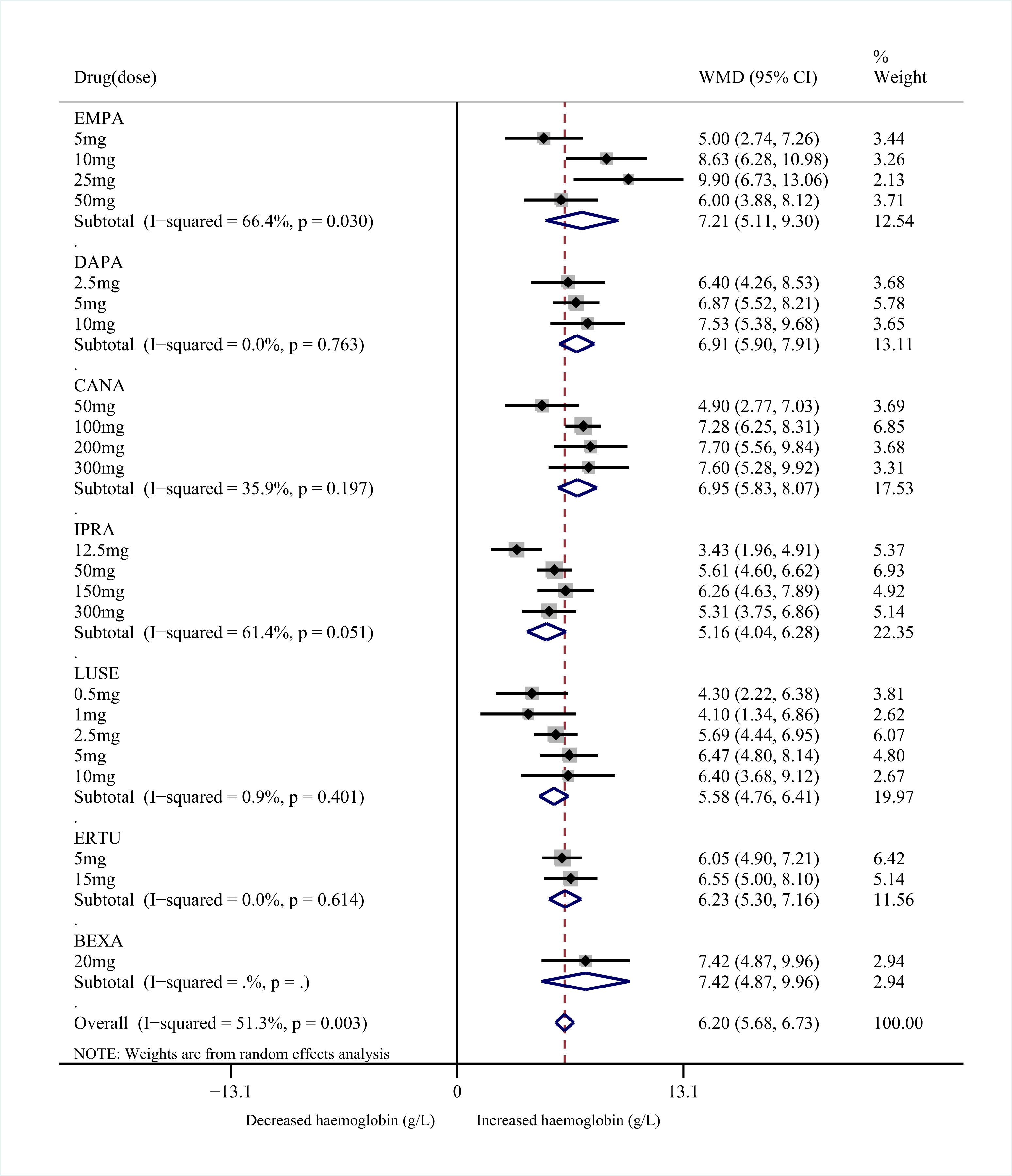


**Figure S1** Meta-analysis of WMD and 95% CI of changes in haemoglobin (g/L) level for SGLT2 inhibitors, stratified by drug. The WMD of each dose was a combined result of multiple observations. WMDs are from a random-effects model analysis. CI: confidence interval; WMD: weighted mean difference.


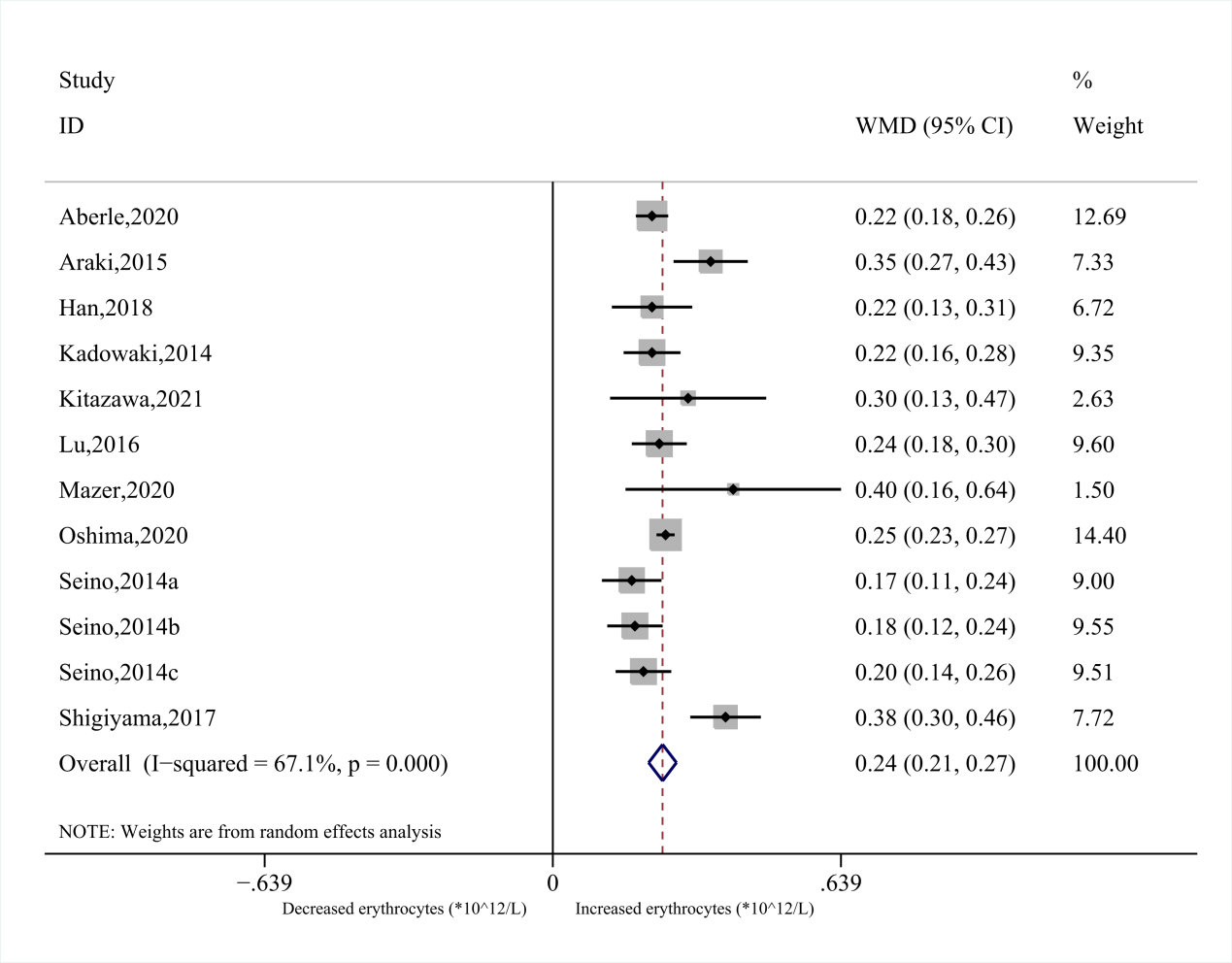


**Figure S2** Meta-analysis of WMD and 95% CI of changes in erythrocyte (*10^12^/L) levels for SGLT2 inhibitors. WMDs are from a random-effects model analysis. CI: confidence interval; WMD: weighted mean difference.


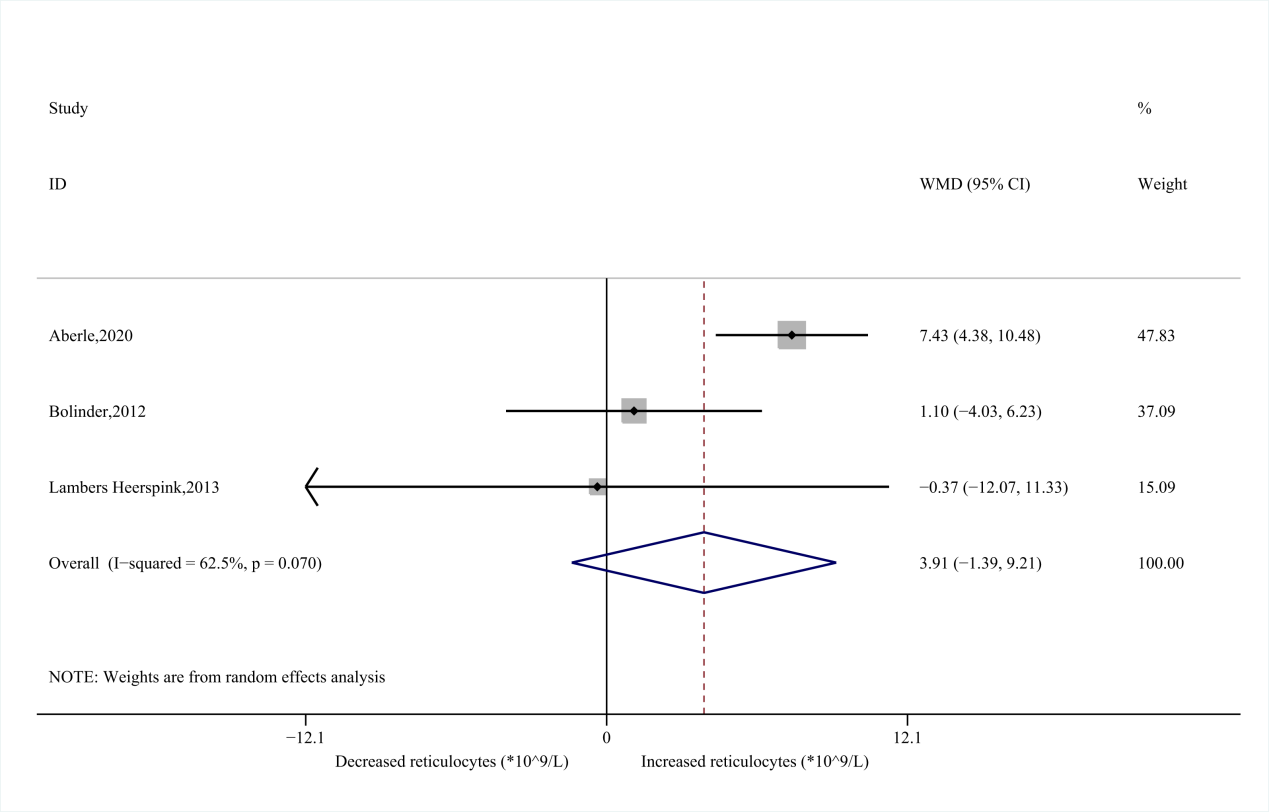


**Figure S3** Meta-analysis of WMD and 95% CI of changes in reticulocyte (*10^9^/L) levels for SGLT2 inhibitors. WMDs are from a random-effects model analysis. CI: confidence interval; WMD: weighted mean difference.


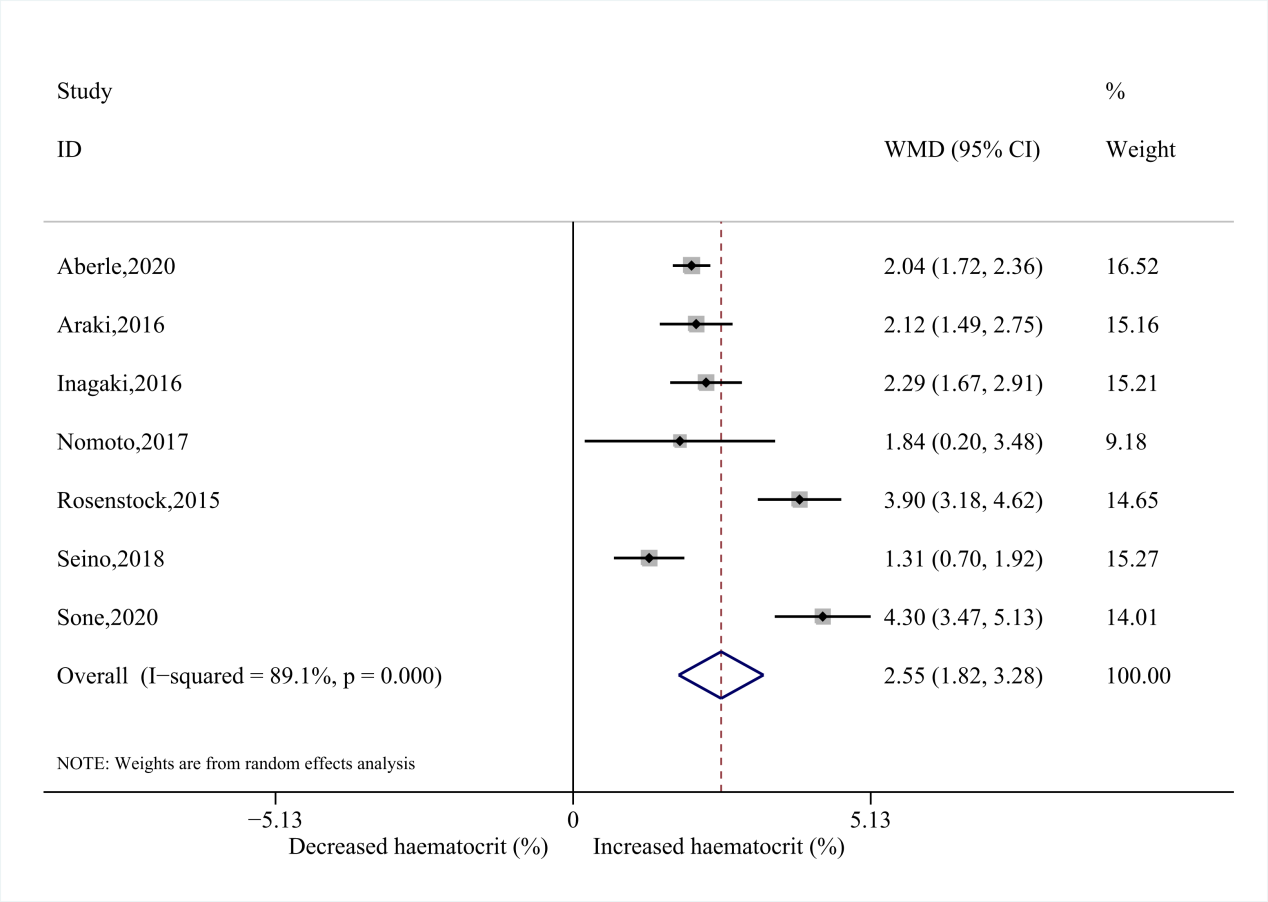


**Figure S4** Meta-analysis of WMD and 95% CI of changes in haematocrit (%) levels for SGLT2 inhibitors combined with insulin therapy. WMDs are from a random-effects model analysis. CI: confidence interval; WMD: weighted mean difference.


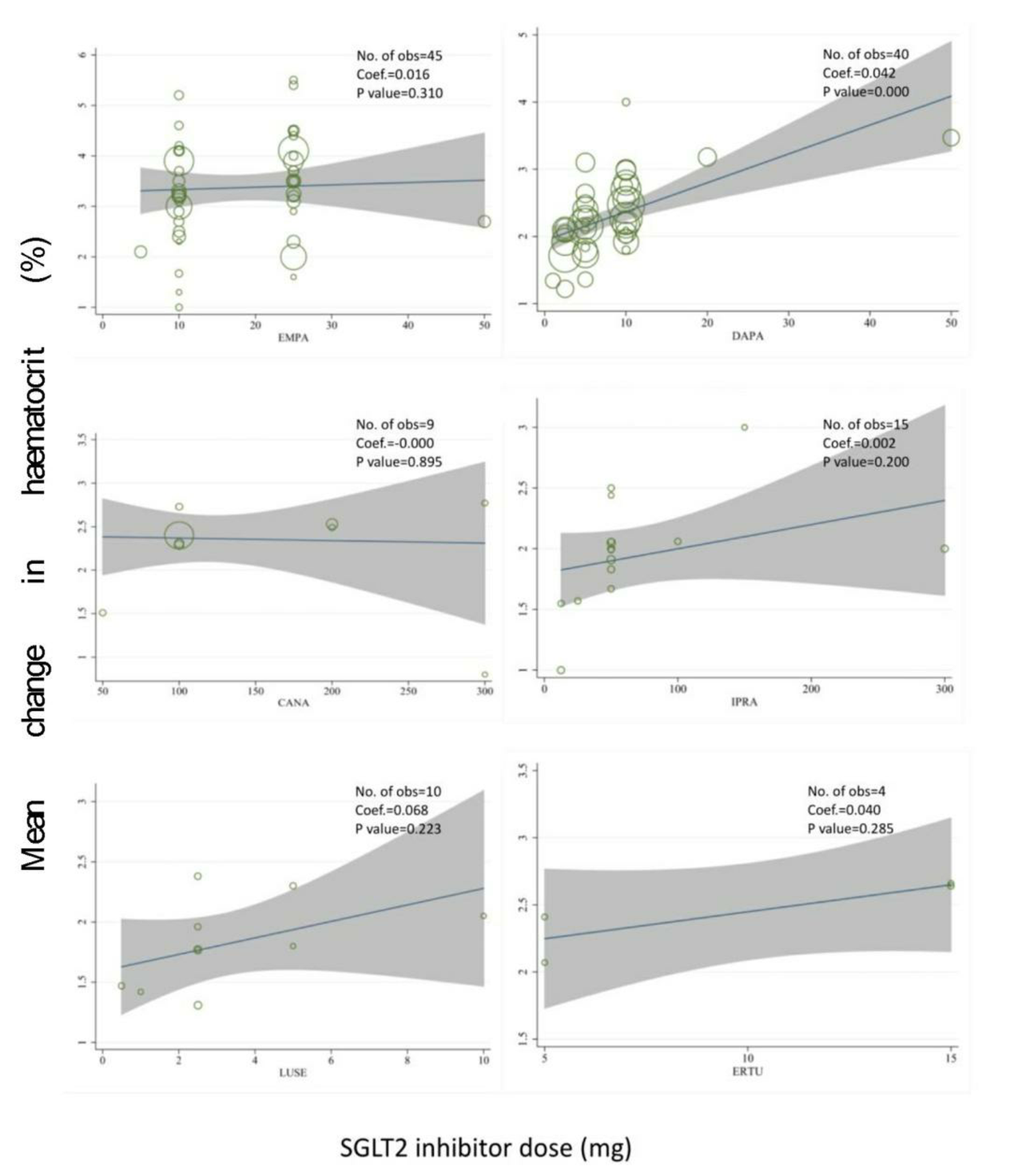


**Figure S5** Meta-regression plots of the association between mean changes in haematocrit levels with various doses of individual SGLT2 inhibitors. Except for dapagliflozin, which exhibited a dose-dependent relationship with haematocrit level (*P* = 0.000), no notable relationship was showed between the haematocrit-increasing effect of individual SGLT2 inhibitors and the various doses (*P* > 0.05).


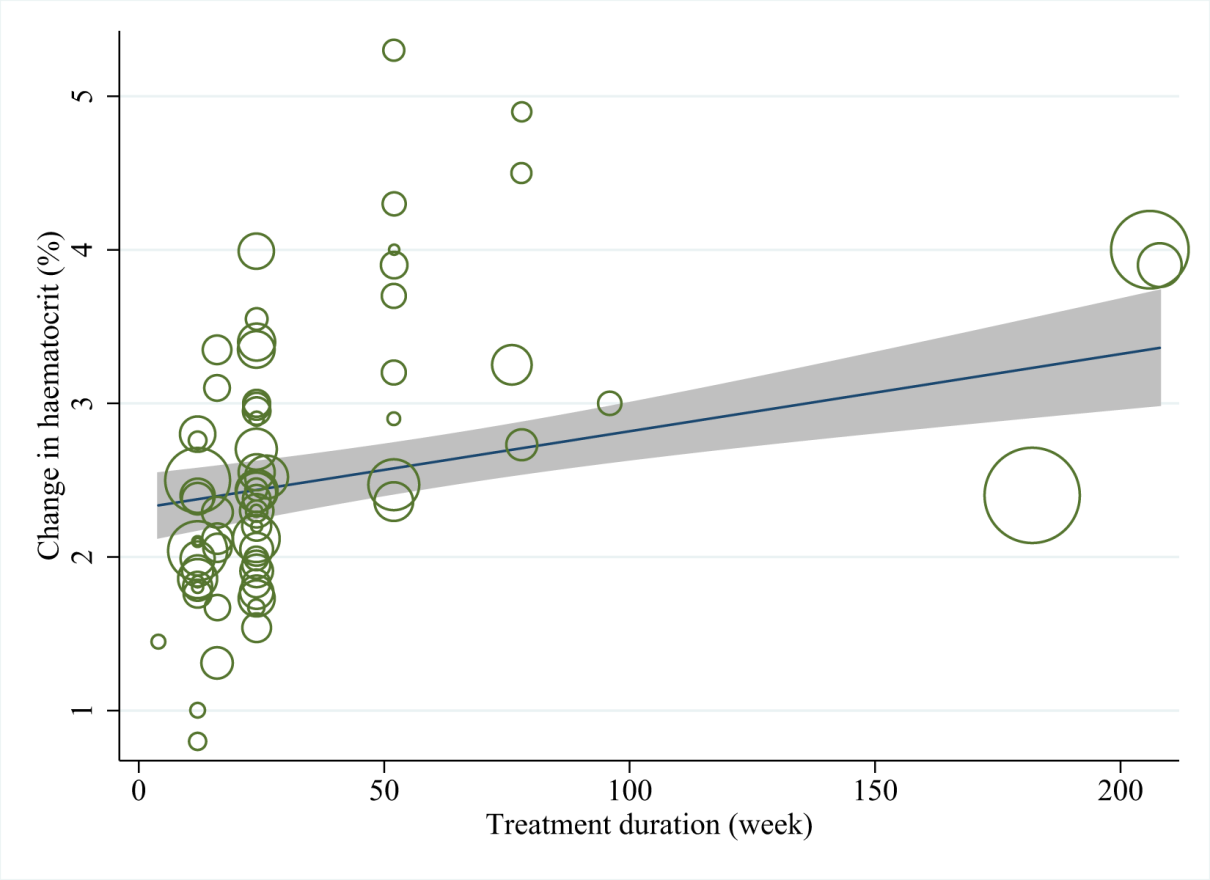


No. of obs=72

I square= test=81.92%

Coef.=0.009, 95%CI [0.005, 0.013]

*P* value=0.000

**Figure S6** Meta-regression plots of the association between mean changes in haematocrit levels and the duration of SGLT2 inhibitor therapy. Irrespective of the type of SGLT2 inhibitor, meta-regression was conducted to reveal the association between therapy effect and duration (coef. = 0.009, 95% CI [0.005, 0.013], *P* = 0.000), showing that the increase in the mean change in haematocrit could be sustained or even slightly increased with long-term therapy.

a.


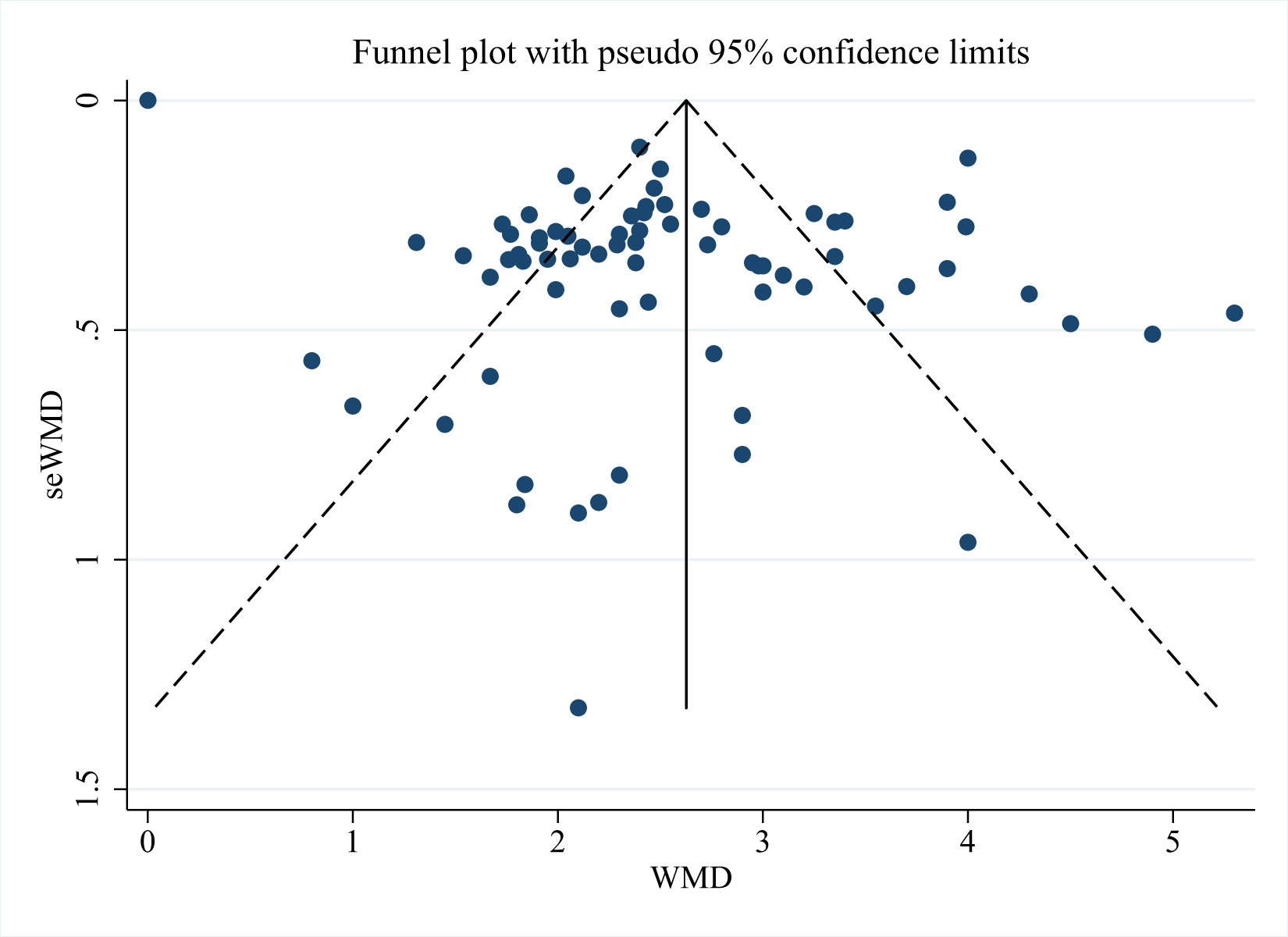


b.


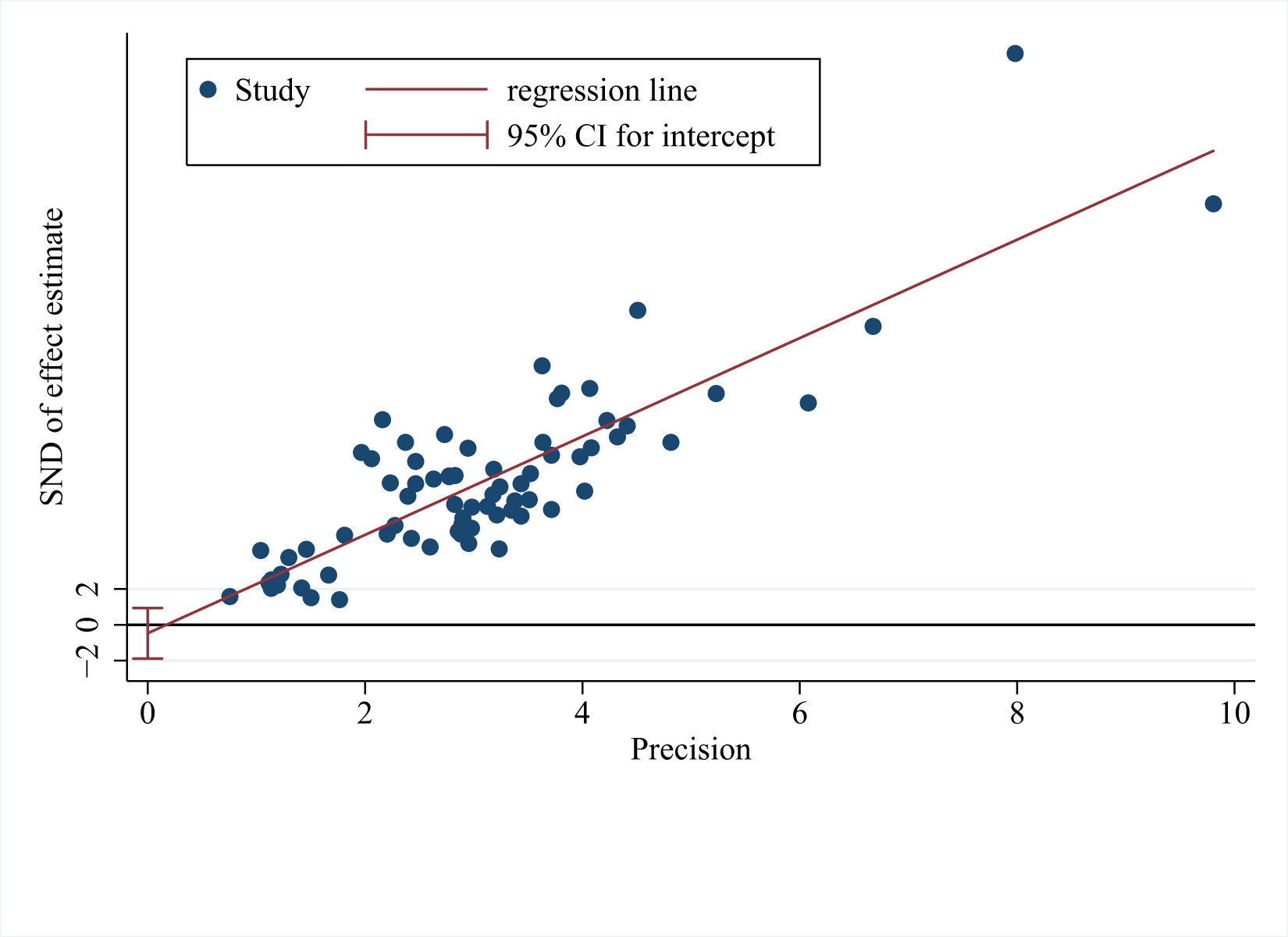


**Figure S7** a. Funnel plot. b. Egger's test graph. A symmetrical funnel plot revealed no potential publication bias for the comparison of haematocrit levels between the intervention and control groups, which was confirmed by Egger's test (*P* = 0.505).
