# Supplementary material for: Effects of SGLT2 inhibitors on haematocrit and haemoglobin levels and the associated cardiorenal benefits in T2DM patients: A meta‐analysis
Source: J Cell Mol Med. 2021 Dec 8;26(2):540–7. doi: 10.1111/jcmm.17115 (PMC8814934; doi:10.1111/jcmm.17115)
Supplement: Supplementary file 16 — Table S1 [file JCMM-26-540-s007.docx]

**Table S1** Search strategy.

| **Data source** | **Search strategy** |
| --- | --- |
| PubMed | #1 sodium-glucose transporter 2 [MeSH Terms] OR sodium-glucose transporter 2 inhibitors [MeSH Terms]  #2 sodium-glucose transporter 2 OR sodium glucose cotransporter 2 OR SGLT2 OR SGLT-2 OR sodium-glucose transporter 2 inhibitors OR sodium glucose cotransporter 2 inhibitors OR SGLT2 inhibitors OR SGLT-2 inhibitors OR SGLT2i OR SGLT-2i OR dapagliflozin OR farxiga OR bms-512148 OR canagliflozin OR invokana OR jnj-28431754 OR empagliflozin OR jardiance OR bi10773 OR ertugliflozin OR steglatro OR ipragliflozin OR suglat OR asp1941 OR luseogliflozin OR lusefi OR TS-071 OR tofogliflozin OR apleway OR deberza OR ertugliflozin OR pf04971729 OR bexagliflozin OR egt0001442 OR remogliflozin OR remogliflozin etabonate OR ta-7284 OR sergliflozin etabonate OR sergliflozin OR shr3824 OR gsk-1614235 OR sotagliflozin OR lx4211 OR zynquista: [TIAB]  #3 anaemia OR hemoglobin OR haemoglobin OR hematocrit OR haematocrit OR Hct OR red blood cell count OR RBC count OR erythrocyte concentration OR reticulocyte OR EPO OR erythropoietin OR erythropoiesis OR hematopoiesis: [TW]  #4 Diabetes Mellitus, Type 2 [MeSH Terms] OR T2DM [TIAB] OR type 2 diabetes mellitus [TIAB]  #5 (((randomized controlled trial [PT]) OR (controlled clinical trial [PT]) OR (clinical trial [PT]) OR (randomized [TIAB] OR randomized [TIAB]) OR (placebo [TIAB]) OR (drug therapy [SH]) OR (randomly [TIAB]) OR (trial [TIAB]) OR (groups [TIAB])) NOT (animals [MH] NOT humans [MH]))  #6 (#1 OR #2) AND #3 AND #4 AND #5 |
| EMBASE | #1 sodium-glucose transporter 2 OR sodium glucose cotransporter 2 OR SGLT2 OR SGLT-2 OR sodium-glucose transporter 2 inhibitors OR sodium glucose cotransporter 2 inhibitors OR SGLT2 inhibitors OR SGLT-2 inhibitors OR SGLT2i OR SGLT-2i OR dapagliflozin OR farxiga OR bms-512148 OR canagliflozin OR invokana OR jnj-28431754 OR empagliflozin OR jardiance OR bi10773 OR ertugliflozin OR steglatro OR ipragliflozin OR suglat OR asp1941 OR luseogliflozin OR lusefi OR TS-071 OR tofogliflozin OR apleway OR deberza OR ertugliflozin OR pf04971729 OR bexagliflozin OR egt0001442 OR remogliflozin OR remogliflozin etabonate OR ta-7284 OR sergliflozin etabonate OR sergliflozin OR shr3824 OR gsk-1614235 OR sotagliflozin OR lx4211 OR zynquista: [TI AB KW]  #2 anaemia OR hemoglobin OR haemoglobin OR hematocrit OR haematocrit OR Hct OR red blood cell count OR RBC count OR erythrocyte concentration OR reticulocyte OR EPO OR erythropoietin OR erythropoiesis OR hematopoiesis: [Quick search]  #3 T2DM OR type 2 diabetes mellitus OR type 2 diabetes: [TI AB KW]  #4 (crossover AND ('procedure'/exp OR procedure)) OR (double AND ('blind'/exp OR blind) AND ('procedure'/exp OR procedure)) OR (randomized AND controlled AND ('trial'/exp OR trial)) OR ('single blind' AND ('procedure'/exp OR procedure)) OR (random* OR factorial* OR crossover* OR (cross AND over*)) OR placebo* OR (doubl* AND adj AND blind*) OR (singl* AND adj AND blind*) OR assign* OR allocat* OR volunteer*  #5 #1 AND #2 AND #3 AND #4 |
| CENTRAL | #1 Sodium-Glucose Transporter 2 Inhibitors [MeSH]  #2 sodium-glucose transporter 2 OR sodium glucose cotransporter 2 OR SGLT2 OR SGLT-2 OR sodium-glucose transporter 2 inhibitors OR sodium glucose cotransporter 2 inhibitors OR SGLT2 inhibitors OR SGLT-2 inhibitors OR SGLT2i OR SGLT-2i OR dapagliflozin OR farxiga OR bms-512148 OR canagliflozin OR invokana OR jnj-28431754 OR empagliflozin OR jardiance OR bi10773 OR ertugliflozin OR steglatro OR ipragliflozin OR suglat OR asp1941 OR luseogliflozin OR lusefi OR TS-071 OR tofogliflozin OR apleway OR deberza OR ertugliflozin OR pf04971729 OR bexagliflozin OR egt0001442 OR remogliflozin OR remogliflozin etabonate OR ta-7284 OR sergliflozin etabonate OR sergliflozin OR shr3824 OR gsk-1614235 OR sotagliflozin OR lx4211 OR zynquista: [TI AB KW]  #3 anaemia OR hemoglobin OR haemoglobin OR hematocrit OR haematocrit OR Hct OR red blood cell count OR RBC count OR erythrocyte concentration OR reticulocyte OR EPO OR erythropoietin OR erythropoiesis OR hematopoiesis: [All Text]  #4 T2DM OR type 2 diabetes mellitus OR type 2 diabetes: [TI AB KW]  #5 [Trials]  #6 (#1 OR #2) AND #3 AND #4 AND #5 |
| Web of Science | #1 sodium-glucose transporter 2 OR sodium glucose cotransporter 2 OR SGLT2 OR SGLT-2 OR sodium-glucose transporter 2 inhibitors OR sodium glucose cotransporter 2 inhibitors OR SGLT2 inhibitors OR SGLT-2 inhibitors OR SGLT2i OR SGLT-2i OR dapagliflozin OR farxiga OR bms-512148 OR canagliflozin OR invokana OR jnj-28431754 OR empagliflozin OR jardiance OR bi10773 OR ertugliflozin OR steglatro OR ipragliflozin OR suglat OR asp1941 OR luseogliflozin OR lusefi OR TS-071 OR tofogliflozin OR apleway OR deberza OR ertugliflozin OR pf04971729 OR bexagliflozin OR egt0001442 OR remogliflozin OR remogliflozin etabonate OR ta-7284 OR sergliflozin etabonate OR sergliflozin OR shr3824 OR gsk-1614235 OR sotagliflozin OR lx4211 OR zynquista: [TS]  #2 anaemia OR hemoglobin OR haemoglobin OR hematocrit OR haematocrit OR Hct OR red blood cell count OR RBC count OR erythrocyte concentration OR reticulocyte OR EPO OR erythropoietin OR erythropoiesis OR hematopoiesis: [ALL]  #3 T2DM OR type 2 diabetes mellitus OR type 2 diabetes: [TS]  #4 randomized controlled trial OR controlled clinical trial OR clinical trial OR randomized OR randomised OR placebo OR randomly OR trial OR RCT: [TS]  #5 #1 AND #2 AND #3 AND #4 |
