# Supplementary material for: Effects of SGLT2 inhibitors on haematocrit and haemoglobin levels and the associated cardiorenal benefits in T2DM patients: A meta‐analysis
Source: J Cell Mol Med. 2021 Dec 8;26(2):540–7. doi: 10.1111/jcmm.17115 (PMC8814934; doi:10.1111/jcmm.17115)
Supplement: Supplementary file 17 — Table S2 [file JCMM-26-540-s010.docx]

**Table S2** Characteristics of included studies

| **Author,year [Reference]** | **Registration** | | **Design** | **Primary race** | **Study duration (week)** | **Treatment**  **(mg QD)** | **Control** | **N** | | | | **Baseline parameters** | | | | | | | | |
| --- | --- | --- | --- | --- | --- | --- | --- | --- | --- | --- | --- | --- | --- | --- | --- | --- | --- | --- | --- | --- |
|  |  | |  |  |  |  |  |  | | | | **Age (year)** | **Sex (male %)** | **Duration of diabetes (year)** | **HbA1c**  **(%)** | **BMI**  **(kg/m2)** | **eGFR**  **(ml/min/1.73 m2)** | | **Blood pressure**  **(mmHg)** | **HCT(%)** |
| **Empagliflozin** |  | |  |  |  |  |  |  | | | |  |  |  |  |  |  | |  |  |
| Mazer,2020[^1^](#_ENREF_1) | NCT02998970 | | R, DB, PC | W | 26 | 10 | PLA | 97 | | | | 62.8 | 93 | 11 | 7.9 | 27.6 | 88.4 | 139/79 | | 41.96 |
| Ridderstråle,2018[^2^](#_ENREF_2) | NCT01167881 | | MC, R, DB, AC | W | 208 | 25 | GLIM | 1545 | | | | 55.9 | 55 | 1-5 | 7.92 | 30.2 | NR | 133.5/79.4 | | 42.7 |
| Sone,2020[^3^](#_ENREF_3) | NCT02589639 | | MC, R, DB, PC | A | 52 | 10, 25 | PLA | 266 | | | | 58.7 | 72.6 | 13.8 | 8.8 | 26.9 | 84.2 | 135.4/79.9 | | 45.56 |
| Hiruma,2021[^4^](#_ENREF_4) | UMIN000026340A | | R, OL, AC | A | 12 | 10 | SITA | 42 | | | | 50.3 | 73.8 | 3.5 | 7.1 | 29.3 | 86.8 | 143.6/88.3 | | 43.65 |
| Tanaka,2020[^5^](#_ENREF_5) | UMIN000024502 | | MC, R, DB, PC | A | 24 | 10 | PLA | 105 | | | | 64.9 | 68.5 | 13.2 | 7.2 | 26.4 | 68.1 | 133.2/75.7 | | 41.45 |
| Zinman,2015 [^6^](#_ENREF_6) | NCT01131676 | | MC, R, DB, PC | W | 206 | 10, 25 | PLA | 7020 | | | | 63.1 | 71.5 | >10 | 8.1 | 30.6 | 74.1 | 135.5/76.7 | | 41.2 |
| Hadjadj,2016[^7^](#_ENREF_7) | NCT01719003 | | MC, R, DB, AC | W | 24 | 10, 25 | MET | 665 | | | | 52.9 | 53.8 | <1 | 8.69 | 30.4 | 92.5 | 128.3/79.0 | | 45.4 |
| Ross,2015[^8^](#_ENREF_8) | NCT01649297 | | MC, R, DB, PC | W | 16 | 10, 25 | PLA | 535 | | | | 58.3 | 51.8 | >5 | 7.77 | 32 | 89.3 | 131.3/78.7 | | 43.46 |
| Roden,2015[^9^](#_ENREF_9) | NCT01289990 | | MC, R, DB, PC/AC | A | 76 | 10, 25 | PLA/SITA | 899 | | | | 55 | 61 | 1-5 | 7.88 | 28.4 | 87.4 | 131.4/79.1 | | 43.57 |
| Tikkanen,2015[^10^](#_ENREF_10) | NCT01370005 | | MC, R, DB, PC | W | 12 | 10, 25 | PLA | 823 | | | | 60.2 | 60.1 | >10 | 7.9 | 32.6 | 84 | 142.1/83.9 | | 41.33 |
| Lewin,2015[^11^](#_ENREF_11) | NCT01422876 | | MC, R, DB, PC/AC | W | 52 | 10, 25 | PLA/LINA | 667 | | | | 54.6 | 54.6 | 1-5 | 8.02 | 31.6 | 88.9 | 128.3/78.5 | | 43.3 |
| DeFronzo,2015[^12^](#_ENREF_12) | NCT01422876 | | MC, R, DB, PC/AC | W | 52 | 10, 25 | PLA/LINA | 674 | | | | 56.2 | 53.7 | 1-5 | 8 | 31 | 89.6 | 130.1/79.1 | | 41.3 |
| Häring,2013[^13^](#_ENREF_13) | NCT01159600 | | MC, R, DB, PC | A | 24, 76 | 10, 25 | PLA | 666 | | | | 57.1 | 51 | >10 | 8.1 | 28.2 | 87.2 | 128.9/78.6 | | 41.9 |
| Häring,2015[^14^](#_ENREF_14) | NCT01289990 | |  |  |  |  |  |  |  |  |  |  |  |  |  |  |  |  |  |  |
| Ferrannini, 2013a[^15^](#_ENREF_15) | NCT00881530 | | MC, R, OL, AC | W | 78 | 10, 25 | MET | 271 | | | | 58.8 | 49.44 | 1-5 | 7.99 | 28.52 | 92.23 | 131.3/79.8 | | 44.44 |
| Ferrannini, 2013b[^15^](#_ENREF_15) | NCT00881530 | | MC, R, OL, AC | W | 78 | 10, 25 | MET+SITA | 388 | | | | 60 | 51.54 | >5 | 7.91 | 30.21 | 92.85 | 134.7/80.7 | | 43.54 |
| Häring,2014[^16^](#_ENREF_16)  Merker,2015[^17^](#_ENREF_17) | NCT01159600  NCT01289990 | | MC, R, DB, PC | W | 24, 76 | 10, 25 | PLA | 637 | | | | 55.7 | 57 | 1-5 | 7.9 | 29.2 | 89 | 129.4/78.7 | | 42.14 |
|  |  | |  |  |  |  |  |  |  |  |  |  |  |  |  |  |  |  |  |  |
| Rosenstock, 2014[^18^](#_ENREF_18) | NCT01306214 | | MC, R, DB, PC | W | 52 | 10, 25 | PLA | 563 | | | | 56.7 | 45 | >10 | 8.34 | 34.8 | 84 | 133.3/78.8 | | 42.5 |
| Kadowaki,2014[^19^](#_ENREF_19) | NCT01193218 | | MC, R, DB, PC | A | 12 | 5, 10, 25, 50 | PLA | 547 | | | | 57.5 | 75 | NR | 7.95 | 25.5 | 85.7 | 129.2/78.7 | | 45.46 |
| Kovacs,2014[^20^](#_ENREF_20)  Kovacs,2015[^21^](#_ENREF_21) | NCT01210001 | | MC, R, DB, PC | A | 24，76 | 10, 25 | PLA | 498 | | | | 54.5 | 48.4 | 1-5 | 8.1 | 29.2 | 85.7 | 126.1/76.9 | | 40.93 |
|  |  |  |  |  |  |  |  |  |  |  |  |  |  |  |  |  |  |  |  |  |
| Rosenstock, 2015[^22^](#_ENREF_22) | NCT01011868 | | MC, R, DB, PC | W | 78 | 10, 25 | PLA | 494 | | | | 58.8 | 56 | >5 | 8.2 | 32.2 | 84 | 133.0/78.3 | | 41.43 |
| Araki,2015[^23^](#_ENREF_23) | NCT01368081 | | MC, R, OL, AC | A | 52 | 10, 25 | MET | 336 | | | | 61.3 | 72.15 | >10 | 8 | 25.2 | 86.5 | 133.4/77.4 | | 42.42 |
| Softeland,2017[^24^](#_ENREF_24) | NCT01734785 | | MC, R, DB, PC | W | 24 | 10, 25 | PLA | 327 | | | | 55.2 | 60.26 | 5-10 | 7.97 | 30.2 | 92.3 | 130.5/79.2 | | 42.36 |
| Nishimura, 2015[^25^](#_ENREF_25) | NCT01947855 | | MC, R, DB, PC | A | 4 | 10, 25 | PLA | 60 | | | | 62.7 | 78.35 | 5-10 | 7.9 | 24.3 | 80 | 120.9/72.4 | | 43.05 |
| Ferdinand,2019[^26^](#_ENREF_26) | NCT02182830 | | MC, R, DB, PC | African Americans | 24 | Titration, 10→ 25 | PLA | 150 | | | | 56.8 | 52.7 | 9.3 | 8.59 | 35.6 | 91.31 | 148.63/88.35 | | 41.76 |
| **Dapagliflozin** |  | |  |  |  |  |  |  | | | |  |  |  |  |  |  |  | |  |
| Aberle,2020[^27^](#_ENREF_27) | NCT00673231 | | MC, R, DB, PC | W | 12 | 2.5, 5, 10 | PLA | 807 | | | | 59.3 | 47.7 | 13.6 | 8.53 | 33.12 | 78.4 | 138.5/80.1 | | 41.29 |
| Nauck,2011[^28^](#_ENREF_28) | NCT00660907 | | MC, R, DB, AC | W | 52 | Titration, 2.5→ 10 | GLIP | 814 | | | | 58 | 58.5 | 6.5 | 7.7 | 31.45 | 90.1 | 133.3/80.6 | | 41.12 |
| Henry,2012b[^29^](#_ENREF_29) | NCT00859898 | | MC, R, DB, PC/AC | W | 24 | 10 | PLA+MET | 638 | | | | 51.6 | 48.2 | 2.1 | 9.1 | NR | NR | 128.7/80.2 | | 43.36 |
| Henry,2012a[^29^](#_ENREF_29) | NCT00643851 | | MC, R, DB, PC/AC | W | 24 | 5 | PLA+MET | 598 | | | | 51.9 | 55.7 | 1.6 | 9.17 | NR | NR | 127.4/80.0 | | 42.88 |
| Strojek,2011[^30^](#_ENREF_30)  Strojek,2014[^31^](#_ENREF_31) | NCT00680745 | | MC, R, DB, PC | W | 24，48 | 2.5, 5, 10 | PLA | 596 | | | | 59.8 | 48.1 | 7.4 | 8.1 | 29.8 | 76.7 | 133.1/78.9 | | 42.01 |
|  |  |  |  |  |  |  |  |  |  |  |  |  |  |  |  |  |  |  |  |  |
| Bailey, 2010[^32^](#_ENREF_32)  Bailey, 2013[^33^](#_ENREF_33) | NCT00528879 | | MC, R, DB, PC | W | 24,102 | 2.5, 5, 10 | PLA | 546 | | | | 53.9 | 53.2 | 6.1 | 8.05 | 31.5 | NR | 126.8/80.0 | | 42.5 |
| Ferrannini, 2010[^34^](#_ENREF_34)  Bailey, 2015[^35^](#_ENREF_35) | NCT00528372 | | MC, R, DB, PC/AC | W | 24,102 | 2.5, 5, 10 | PLA/MET | 274 | | | | 52.2 | 33 | 0.4 | 8 | 32.6 | 85.1 | NR | | 43.25 |
|  |  |  |  |  |  |  |  |  | | | |  |  |  |  |  |  |  | |  |
| Schumm-Draeger, 2015[^36^](#_ENREF_36) | NCT01217892 | | MC, R, DB, PC | W | 16 | 10 | PLA | 200 | | | | 58.5 | 48 | 5.49 | 7.83 | 31.99 | 83.5 | 132.8/80.5 | | 41.47 |
| Ji, 2014[^37^](#_ENREF_37) | NCT01095653 | | MC, R, DB, PC | A | 12,24 | 5, 10 | PLA | 393 | | | | 51.4 | 65.4 | 1.4 | 8.3 | 25.6 | 92.5 | 123.7/77.4 | | 43.79 |
| List, 2009[^38^](#_ENREF_38) | NCT00263276 | | MC, R, DB, PC/AC | W | 12 | 2.5, 5, 10, 20, 50 | PLA/MET | 389 | | | | 54.2 | 50.3 | NR | 7.8 | 31.7 | NR | 126.4/77.1 | | NR |
| Bailey, 2012[^39^](#_ENREF_39) | NR | | MC, R, DB, PC | W | 24 | 1, 2.5, 5 | PLA | 282 | | | | 53 | 50 | 1.4 | 7.9 | 31.8 | NR | 127.9/79.0 | | 43.22 |
| Araki, 2016[^40^](#_ENREF_40) | NCT02157298 | | MC, R, DB, PC | A | 16 | 5 | PLA | 182 | | | | 58.1 | 70.9 | 14.9 | 8.4 | NR | 78.1 | 131.57 /77.83 | | 43.13 |
| Bolinder, 2012[^41^](#_ENREF_41)  Bolinder, 2014[^42^](#_ENREF_42) | NCT00855166 | | MC, R, DB, PC | W | 24,102 | 10 | PLA | 182 | | | | 60.7 | 55.6 | 5.7 | 7.2 | 31.9 | 84.3 | 134.6/80.5 | | 42.1 |
|  |  |  |  |  |  |  |  |  | | | |  |  |  |  |  |  |  | |  |
| Fioretto,2018[^43^](#_ENREF_43) | NCT02413398 | | MC, R, DB, PC | W | 24 | 10 | PLA | 321 | | | | 65.8 | 56.7 | 14.4 | 8.18 | 32.1 | 53.5 | NR | | NR |
| Pollock,2019[^44^](#_ENREF_44) | NCT02547935 | | MC, R, DB, PC | W | 24 | 10 | PLA | 293 | | | | 64.5 | 71 | 17.91 | 8.4 | 30.45 | 49 | 139.3/76.6 | | 39.99 |
| Lambers Heerspink,2013[^45^](#_ENREF_45) | NCT00976495 | | MC, R, DB, PC/AC | W | 12 | 10 | PLA/ HCTZ | 75 | | | | 56 | 65.3 | 6.3 | 7.5 | NR | 101.1 | NR | | 41.06 |
| Ghanim,2020[^46^](#_ENREF_46) | NCT02433678 | | SC, R, DB, PC | W | 12 | 10 | PLA | 47 | | | | 61 | NR | NR | 6.8 | 38 | 85 | 135/79 | | 41.1 |
| van Bommel,2020[^47^](#_ENREF_47) | NCT02682563 | | SC, R, DB, AC | W | 12 | 10 | GLIC | 44 | | | | 63 | 77 | 10.2 | 7.4 | 31.2 | 87 | 134.9/83.2 | | 40.47 |
| Fuchigami,2020[^48^](#_ENREF_48) | UMIN000028014 | | MC, R, OL, AC | A | 24 | Titration, 5→10 | SITA | 331 | | | | 58 | 60.1 | 6 | 7.8 | 28 | 79 | 133.7/79.8 | | 43.05 |
| Hayashi,2017[^49^](#_ENREF_49) | UMIN000020984 | | SC, R, OL, AC | A | 12 | 5 | SITA | 80 | | | | 54 | 77.5 | 8.3 | 7.58 | 28 | 84.9 | 132.0/87.5 | | 41.65 |
| Kayano,2020[^50^](#_ENREF_50) | UMIN000023834 | | MC, R, DB, AC | A | 24 | 5 | CT | 74 | | | | 67.7 | 89.2 | 8.5 | 7.5 | 25.7 | NR | 140.6/NR | | 42.8 |
| Nomoto,2017[^51^](#_ENREF_51) | UMIN000015033 | | SC, R, OL, AC | A | 12 | 5 | DPP4is | 29 | | | | 61.4 | 58.6 | 15.9 | 7.3 | 26.1 | 71.3 | NR | | 41.23 |
| Phrommintikul,2019[^52^](#_ENREF_52) | NCT03178591 | | SC, R, DB, AC | A | 24 | 10 | VILD | 49 | | | | 63.22 | 53.1 | NR | 8.21 | 25.28 | 69.99 | 124.50/67.97 | | NR |
| Satirapoj,2019[^53^](#_ENREF_53) NR | | | SC, R, OL, AC | A | 12 | 10 | ST | 57 | | | | 57.9 | 43.6 | 8.8 | 8.6 | 28.1 | 88 | 132.9/77.7 | | NR |
| Shigiyama,2017[^54^](#_ENREF_54) | UMIN000018754 | | MC, R, OL, AC | A | 16 | 5 | MET | 74 | | | | 58.7 | 63.6 | 5.9 | 6.9 | 26.6 | NR | 129.6/80.9 | | 42.65 |
| Singh,2020[^55^](#_ENREF_55) | NCT02397421 | | SC, R, DB, PC | W | 52 | 10 | PLA | 56 | | | | 67.1 | 66.1 | 8.77 | 7.7 | 32.5 | 72 | 133.9/72.7 | | NR |
| Lee,2020[^56^](#_ENREF_56) | NCT02459353 | | MC, R, DB, PC | A | 6,12 | 10 | PLA | 84 | | | | 58.7 | 41.7 | 15.1 | 8.27 | 26.9 | 86.3 | 123.3/77 | | NR |
| Cho,2019[^57^](#_ENREF_57) | UMIN000022804 | | MC, R, OL, AC | A | 24 | 5 | PIOG | 71 | | | | 63.3 | 59.2 | >10 | 6.9 | 28.6 | 69.8 | 130.1/76.6 | | 42.36 |
| **Canagliflozin** | | | | | | | | | | | | | | | | | | | |  |
| Rosenstock, 2012[^58^](#_ENREF_58) | | NCT00642278 | MC, R, DB, PC/AC | NR | 12 | 50, 100, 200, 300 | PLA/SITA | 387 | | | | 52.5 | 53 | 6 | 7.75 | 31.5 | NR | 127/78 | | NR |
| Sha, 2014[^59^](#_ENREF_59) | | NCT01483781 | SC, R, DB, PC | W | 12 | 300 | PLA | 36 | 62.8 | | | | 86 | 8.5 | 7.65 | 30.3 | 97.3 | 132.9/80.0 | | 41.6 |
| Oshima,2020[^60^](#_ENREF_60) | | NCT02065791 | MC, R, DB, PC | W | 182 | 100 | PLA | 4401 | | 63 | | | 66.1 | 15.8 | 8.3 | 31.3 | 56.2 | 140.0/78.3 | | 40.4 |
| Inagaki,2014[^61^](#_ENREF_61) | | NCT01413204 | MC, R, DB, PC | A | 24 | 100, 200 | PLA | 271 | | | 58 | | 70.5 | 5.41 | 8.02 | 25.62 | 84.4 | 127.85/77.84 | | NR |
| Inagaki,2016[^62^](#_ENREF_62) | | NCT02220920 | R, DB, PC | A | 16 | 100 | PLA | 146 | | | 58 | | 63.7 | 13.82 | 8.87 | 26.45 | 84.9 | 133.54/77.81 | | 43.18 |
| Takashima,2018[^63^](#_ENREF_63) | | UMIN000031454 | SC, R, OL, AC | NR | 52 | 100 | CT | 40 | | | 65.05 | | 57.5 | NR | 7.4 | 25.2 | 56.3 | 137.5/78.5 | | NR |
| **Ipragliflozin** | | | | | | | | | | | | | | | | | | | |  |
| Kashiwagi, 2014a[^64^](#_ENREF_64) | | NCT01057628 | MC, R, DB, PC | A | 16 | 50 | PLA | 129 | | | 59.4 | | 69.7 | 6.7 | 8.3 | 25.5 | 87.8 | 130.0/128.2 | | 42.82 |
| Kashiwagi, 2014b[^65^](#_ENREF_65) | | NCT00621868 | MC, R, DB, PC | A | 12 | 12.5, 25, 50, 100 | PLA | 360 | | | 60 | | 64.7 | 6.7 | 8.3 | 25.6 | NR | NR | | NR |
| Kashiwagi,2014c[^66^](#_ENREF_66) | | NCT01242215 | MC, R, DB, PC | A | 24 | 50 | PLA | 240 | | | | 59.7 | 65.9 | 10.45 | 8.37 | 25.3 | 84.75 | 130/76.6 | | 43.7 |
| Kashiwagi,2014d[^67^](#_ENREF_67) | | NCT01225081 | MC, R, DB, PC | A | 24 | 50 | PLA | 151 | | | | 56.2 | 74.2 | 6.8 | 8.29 | 27.1 | 90.96 | 130.4/77.9 | | 43.34 |
| Kashiwagi,2015[^68^](#_ENREF_68) | | NCT01316094 | MC, R, DB, PC | A | 24 | 50 | PLA | 164 | | | | 64.4 | 78.1 | 9.5 | 7.54 | 25.59 | 60.9 | 133.3/77.2 | | 40.99 |
| Fonseca,2013[^69^](#_ENREF_69) | | NCT01071850 | MC, R, DB, AC/PC | W | 12 | 12.5, 50, 150, 300 | PLA/MET | 411 | | | | 53.7 | 51.3 | 4.6 | 7.9 | 30.9 | NR | NR | | NR |
| Wilding,2013[^70^](#_ENREF_70) | | NCT01117584 | MC, R, DB, PC | W | 12 | 12.5, 50, 150, 300 | PLA | 342 | | | | 57.4 | 51.2 | 5.9 | 7.8 | 31.7 | NR | NR | | NR |
| Lu, 2016[^71^](#_ENREF_71) | | NCT01505426 | MC, R, DB, PC | A | 24 | 50 | PLA | 170 | | | | 53.7 | 38.6 | 6.16 | 7.7 | 26.8 | 149.3 | NR | | NR |
| Han,2018[^72^](#_ENREF_72) | | NCT02452632 | MC, R, DB, PC | A | 24 | 50 | PLA | 142 | | | | 57.53 | 49.7 | 11.48 | 7.91 | 25.76 | 89.99 | NR | | NR |
| Kitazawa,2021[^73^](#_ENREF_73) | | jRCTs031180205 | MC, R, OL, AC | A | 52 | 50 | SITA | 111 | | | | 59.2 | 61.3 | 9.7 | 7.54 | 26.6 | NR | 135.5/81.9 | | 41.89 |
| **Luseogliflozin** | | | | | | | | | | | | | | | | | | | |  |
| Seino, 2014a[^74^](#_ENREF_74) | | JapicCTI-101191 | MC, R, DB, PC | A | 12 | 1, 2.5, 5, 10 | PLA | 280 | | | | 58 | 70.4 | 5 | 7.9 | 24.83 | NR | 127.2/76.3 | | 42.63 |
| Seino,2014b[^75^](#_ENREF_75) | | JapicCTI-090908 | MC, R, DB, PC | A | 12 | 0.5, 2.5, 5 | PLA | 236 | | | | 57 | 67.8 | 6 | 8.1 | 24.9 | NR | 124.4/74.5 | | 42.03 |
| Seino,2014c[^76^](#_ENREF_76) | | JapicCTI-111661 | MC, R, DB, PC | A | 24 | 2.5 | PLA | 158 | | | | 59.3 | 73.4 | 6.3 | 8.2 | 25.66 | NR | 129.0/76.8 | | 42.39 |
| Haneda,2016[^77^](#_ENREF_77) | | JapicCTI-111543 | R, DB, PC | A | 24 | 2.5 | PLA | 145 | | | | 68 | 76.6 | 11.1 | 7.71 | 25.58 | 52.1 | 131.3/75.4 | | 40.05 |
| Seino,2018[^78^](#_ENREF_78) | | JapicCTI-142582 | MC, R, DB, PC | A | 16 | 2.5 | PLA | 233 | | | | 57.3 | 69.9 | 11.8 | 8.74 | 25.33 | 86.9 | 128.2/76.0 | | 43.2 |
| **Ertugliflozin** | | | | | | | | | | | | | | | | | | | |  |
| Gallo,2019[^79^](#_ENREF_79) | | NCT02033889 | MC, R, DB, PC/AC | W | 52,104 | 5, 15 | PLA/GLIM | 621 | | | | 56.6 | 46.4 | 7.99 | 8.12 | 31.1 | 90.5 | 130.1/78.0 | | 42.1 |
| Terra,2017[^80^](#_ENREF_80)  Aronson,2018[^81^](#_ENREF_81) | | NCT01958671 | MC, R, DB, PC/AC | W | 26,52 | 5, 15 | PLA/MET | 461 | | | | 56.4 | 56.6 | 4.99 | 8.21 | 33 | 87.7 | NR | | 43.23 |
|  | |  |  |  |  |  |  |  | | | |  |  |  |  |  |  |  | |  |
| Ji,2019[^82^](#_ENREF_82) | | NCT02630706 | MC, R, DB, PC | A | 26 | 5, 15 | PLA | 506 | | | | 56.5 | 55.5 | 7 | 8.1 | 26 | 99.3 | NR | | NR |
| **Bexagliflozin** | |  |  |  |  |  |  |  |  | | | |  |  |  |  |  |  | |  |
| Halvorsen,2019a[^83^](#_ENREF_83) | | NCT01377844 | MC, R, DB, PC | W | 96 | 20 | PLA | 283 | | | | 55.6 | 41 | 7.47 | NR | 30.1 | NR | 127.2/76.9 | | 45.0 |
| Halvorsen,2019b[^84^](#_ENREF_84) | | NCT03115112 | MC, R, DB, AC | W | 24 | 20 | SITA | 384 | | | | 59.4 | 64.1 | 8.79 | 7.99 | 31.7 | NR | 135.3/81 | | 42.15 |

Abbreviations: HbA1c, glycated hemoglobin; BMI, body weight index; eGFR, estimated glomerular filtration rate; MC, multicenter; SS, single center; R, randomized; OL, open label; DB, double blind; PC, placebo control; AC, active control; ST, standard treatment; CT, conventional treatment; W, white; A, Asian; PLA, placebo; MET, metformin; DPP4is, Dipeptidyl-Peptidase-4 inhibitors; SITA, sitagliptin; LINA, linagliptin; VILD, vildagliptin; GLIP, glipzide; GLIC, gliclazide; GLIM, glimepiride; PIOG, pioglitazone; HCTZ, hydrochlorothiazide; NR, not reported.

Reference:

1. Mazer CD, Hare GMT, Connelly PW*, et al.* Effect of empagliflozin on erythropoietin levels, iron stores, and red blood cell morphology in patients with type 2 diabetes mellitus and coronary artery disease. Circulation. 2020; **141**: 704-707.

2. Ridderstråle M, Rosenstock J, Andersen K, Woerle H, Salsali A. Empagliflozin compared with glimepiride in metformin-treated patients with type 2 diabetes: 208-week data from a masked randomized controlled trial. Diabetes Obes Metab. 2018; **20**: 2768-2777.

3. Sone H, Kaneko T, Shiki K*, et al.* Efficacy and safety of empagliflozin as add-on to insulin in Japanese patients with type 2 diabetes: A randomized, double-blind, placebo-controlled trial. Diabetes Obes Metab. 2020; **22**: 417-426.

4. Hiruma S, Shigiyama F, Hisatake S*, et al.* A prospective randomized study comparing effects of empagliflozin to sitagliptin on cardiac fat accumulation, cardiac function, and cardiac metabolism in patients with early-stage type 2 diabetes: the ASSET study. Cardiovasc Diabetol. 2021; **20**.

5. Tanaka A, Shimabukuro M, Machii N, Teragawa H, Okada Y, Shima KR. Secondary analyses to assess the profound effects of empagliflozin on endothelial function in patients with type 2 diabetes and established cardiovascular diseases: The placebo-controlled double-blind randomized effect of empagliflozin on endothelial function in cardiovascular high risk diabetes mellitus: Multi-center placebo-controlled double-blind randomized trial. J Diabetes Investig. 2020; **11**: 1551-1563.

6. Zinman B, Wanner C, Lachin JM*, et al.* Empagliflozin, cardiovascular outcomes, and mortality in type 2 diabetes. N Engl J Med. 2015; **373**: 2117-2128.

7. Hadjadj S, Rosenstock J, Meinicke T, Woerle HJ. Initial combination of empagliflozin and metformin in patients with type 2 diabetes. Diabetes Care. 2016; **39**: 1718-1728.

8. Ross S, Thamer C, Cescutti J, Meinicke T, Woerle HJ, Broedl UC. Efficacy and safety of empagliflozin twice daily versus once daily in patients with type 2 diabetes inadequately controlled on metformin: a 16-week, randomized, placebo-controlled trial. Diabetes Obes Metab. 2015; **17**: 699-702.

9. Roden M, Merker L, Christiansen AV*, et al.* Safety, tolerability and effects on cardiometabolic risk factors of empagliflozin monotherapy in drug-naïve patients with type 2 diabetes: a double-blind extension of a Phase III randomized controlled trial. Cardiovasc Diabetol. 2015; **14**: 154.

10. Tikkanen I, Narko K, Zeller C*, et al.* Empagliflozin reduces blood pressure in patients with type 2 diabetes and hypertension. Diabetes Care. 2015; **38**: 420-428.

11. Lewin A, DeFronzo RA, Patel S*, et al.* Initial combination of empagliflozin and linagliptin in subjects with type 2 diabetes. Diabetes Care. 2015; **38**: 394-402.

12. DeFronzo RA, Lewin A, Patel S*, et al.* Combination of empagliflozin and linagliptin as second-line therapy in subjects with type 2 diabetes inadequately controlled on metformin. Diabetes Care. 2015; **38**: 384-393.

13. Häring HU, Merker L, Seewaldt-Becker E*, et al.* Empagliflozin as add-on to metformin plus sulfonylurea in patients with type 2 diabetes: a 24-week, randomized, double-blind, placebo-controlled trial. Diabetes Care. 2013; **36**: 3396-3404.

14. Haering HU, Merker L, Christiansen AV*, et al.* Empagliflozin as add-on to metformin plus sulphonylurea in patients with type 2 diabetes. Diabetes Res Clin Pract. 2015; **110**: 82-90.

15. Ferrannini E, Berk A, Hantel S*, et al.* Long-term safety and efficacy of empagliflozin, sitagliptin, and metformin: an active-controlled, parallel-group, randomized, 78-week open-label extension study in patients with type 2 diabetes. Diabetes Care. 2013; **36**: 4015-4021.

16. Häring HU, Merker L, Seewaldt-Becker E*, et al.* Empagliflozin as add-on to metformin in patients with type 2 diabetes: a 24-week, randomized, double-blind, placebo-controlled trial. Diabetes Care. 2014; **37**: 1650-1659.

17. Merker L, Häring HU, Christiansen AV*, et al.* Empagliflozin as add-on to metformin in people with Type 2 diabetes. Diabet Med. 2015; **32**: 1555-1567.

18. Rosenstock J, Jelaska A, Frappin G*, et al.* Improved glucose control with weight loss, lower insulin doses, and no increased hypoglycemia with empagliflozin added to titrated multiple daily injections of insulin in obese inadequately controlled type 2 diabetes. Diabetes Care. 2014; **37**: 1815-1823.

19. Kadowaki T, Haneda M, Inagaki N*, et al.* Empagliflozin monotherapy in Japanese patients with type 2 diabetes mellitus: a randomized, 12-week, double-blind, placebo-controlled, phase II trial. Adv Ther. 2014; **31**: 621-638.

20. Kovacs CS, Seshiah V, Swallow R*, et al.* Empagliflozin improves glycaemic and weight control as add-on therapy to pioglitazone or pioglitazone plus metformin in patients with type 2 diabetes: A 24-week, randomized, placebo-controlled trial. Diabetes Obes Metab. 2014; **16**: 147-158.

21. Kovacs CS, Seshiah V, Merker L*, et al.* Empagliflozin as add-on therapy to pioglitazone with or without metformin in patients with type 2 diabetes mellitus. Clin Ther. 2015; **37**: 1773-1788.

22. Rosenstock J, Jelaska A, Zeller C, Kim G, Broedl UC, Woerle HJ. Impact of empagliflozin added on to basal insulin in type 2 diabetes inadequately controlled on basal insulin: a 78-week randomized, double-blind, placebo-controlled trial. Diabetes Obes Metab. 2015; **17**: 936-948.

23. Araki E, Tanizawa Y, Tanaka Y*, et al.* Long-term treatment with empagliflozin as add-on to oral antidiabetes therapy in Japanese patients with type 2 diabetes mellitus. Diabetes Obes Metab. 2015; **17**: 665-674.

24. Søfteland E, Meier JJ, Vangen B, Toorawa R, Maldonado-Lutomirsky M, Broedl UC. Empagliflozin as add-on therapy in patients with type 2 diabetes inadequately controlled with linagliptin and metformin: a 24-week randomized, double-blind, parallel-group trial. Diabetes Care. 2017; **40**: 201-209.

25. Nishimura R, Tanaka Y, Koiwai K*, et al.* Effect of empagliflozin monotherapy on postprandial glucose and 24-hour glucose variability in Japanese patients with type 2 diabetes mellitus: A randomized, double-blind, placebo-controlled, 4-week study. Cardiovasc Diabetol. 2015; **14**.

26. Ferdinand KC, Izzo JL, Lee J*, et al.* Antihyperglycemic and blood pressure effects of empagliflozin in black patients with type 2 diabetes mellitus and hypertension. Circulation. 2019; **139**: 2098-2109.

27. Aberle J, Menzen M, Schmid SM*, et al.* Dapagliflozin effects on haematocrit, red blood cell count and reticulocytes in insulin-treated patients with type 2 diabetes. Sci Rep. 2020; **10**: 22396.

28. Nauck MA, Del Prato S, Meier JJ*, et al.* Dapagliflozin versus glipizide as add-on therapy in patients with type 2 diabetes who have inadequate glycemic control with metformin: a randomized, 52-week, double-blind, active-controlled noninferiority trial. Diabetes Care. 2011; **34**: 2015-2022.

29. Henry RR, Murray AV, Marmolejo MH, Hennicken D, Ptaszynska A, List JF. Dapagliflozin, metformin XR, or both: initial pharmacotherapy for type 2 diabetes, a randomised controlled trial. Int J Clin Pract. 2012; **66**: 446-456.

30. Strojek K, Yoon KH, Hruba V, Elze M, Langkilde AM, Parikh S. Effect of dapagliflozin in patients with type 2 diabetes who have inadequate glycaemic control with glimepiride: a randomized, 24-week, double-blind, placebo-controlled trial. Diabetes Obes Metab. 2011; **13**: 928-938.

31. Strojek K, Yoon KH, Hruba V, Sugg J, Langkilde AM, Parikh S. Dapagliflozin added to glimepiride in patients with type 2 diabetes mellitus sustains glycemic control and weight loss over 48 weeks: a randomized, double-blind, parallel-group, placebo-controlled trial. Diabetes Ther. 2014; **5**: 267-283.

32. Bailey CJ, Gross JL, Pieters A, Bastien A, List JF. Effect of dapagliflozin in patients with type 2 diabetes who have inadequate glycaemic control with metformin: a randomised, double-blind, placebo-controlled trial. Lancet. 2010; **375**: 2223-2233.

33. Bailey CJ, Gross JL, Hennicken D, Iqbal N, Mansfield TA, List JF. Dapagliflozin add-on to metformin in type 2 diabetes inadequately controlled with metformin: a randomized, double-blind, placebo-controlled 102-week trial. BMC Med. 2013; **11**: 43.

34. Ferrannini E, Ramos SJ, Salsali A, Tang W, List JF. Dapagliflozin monotherapy in type 2 diabetic patients with inadequate glycemic control by diet and exercise: a randomized, double-blind, placebo-controlled, phase 3 trial. Diabetes Care. 2010; **33**: 2217-2224.

35. Bailey CJ, Morales Villegas EC, Woo V, Tang W, Ptaszynska A, List JF. Efficacy and safety of dapagliflozin monotherapy in people with Type 2 diabetes: a randomized double-blind placebo-controlled 102-week trial. Diabet Med. 2015; **32**: 531-541.

36. Schumm-Draeger PM, Burgess L, Korányi L, Hruba V, Hamer-Maansson JE, de Bruin TW. Twice-daily dapagliflozin co-administered with metformin in type 2 diabetes: a 16-week randomized, placebo-controlled clinical trial. Diabetes Obes Metab. 2015; **17**: 42-51.

37. Ji L, Ma J, Li H*, et al.* Dapagliflozin as monotherapy in drug-naive Asian patients with type 2 diabetes mellitus: a randomized, blinded, prospective phase III study. Clin Ther. 2014; **36**: 84-100.

38. List JF, Woo V, Morales E, Tang W, Fiedorek FT. Sodium-glucose cotransport inhibition with dapagliflozin in type 2 diabetes. Diabetes Care. 2009; **32**: 650-657.

39. Bailey CJ, Iqbal N, T'Joen C, List JF. Dapagliflozin monotherapy in drug-naïve patients with diabetes: a randomized-controlled trial of low-dose range. Diabetes Obes Metab. 2012; **14**: 951-959.

40. Araki E, Onishi Y, Asano M*, et al.* Efficacy and safety of dapagliflozin in addition to insulin therapy in Japanese patients with type 2 diabetes: Results of the interim analysis of 16-week double-blind treatment period. J Diabetes Investig. 2016; **7**: 555-564.

41. Bolinder J, Ljunggren Ö, Kullberg J*, et al.* Effects of dapagliflozin on body weight, total fat mass, and regional adipose tissue distribution in patients with type 2 diabetes mellitus with inadequate glycemic control on metformin. J Clin Endocrinol Metab. 2012; **97**: 1020-1031.

42. Bolinder J, Ljunggren Ö, Johansson L*, et al.* Dapagliflozin maintains glycaemic control while reducing weight and body fat mass over 2 years in patients with type 2 diabetes mellitus inadequately controlled on metformin. Diabetes Obes Metab. 2014; **16**: 159-169.

43. Fioretto P, Del Prato S, Buse JB*, et al.* Efficacy and safety of dapagliflozin in patients with type 2 diabetes and moderate renal impairment (chronic kidney disease stage 3A): the DERIVE Study. Diabetes Obes Metab. 2018; **20**: 2532-2540.

44. Pollock C, Stefánsson B, Reyner D*, et al.* Albuminuria-lowering effect of dapagliflozin alone and in combination with saxagliptin and effect of dapagliflozin and saxagliptin on glycaemic control in patients with type 2 diabetes and chronic kidney disease (DELIGHT): a randomised, double-blind, placebo-controlled trial. Lancet Diabetes Endocrinol. 2019; **7**: 429-441.

45. Lambers Heerspink HJ, de Zeeuw D, Wie L, Leslie B, List J. Dapagliflozin a glucose-regulating drug with diuretic properties in subjects with type 2 diabetes. Lancet. 2013; **15**: 853-862.

46. Ghanim H, Abuaysheh S, Hejna J*, et al.* Dapagliflozin suppresses hepcidin and increases erythropoiesis. J Clin Endocrinol Metab. 2020; **105**.

47. van Bommel EJM, Muskiet MHA, van Baar MJB*, et al.* The renal hemodynamic effects of the SGLT2 inhibitor dapagliflozin are caused by post-glomerular vasodilatation rather than pre-glomerular vasoconstriction in metformin-treated patients with type 2 diabetes in the randomized, double-blind RED trial. Kidney Int. 2020; **97**: 202-212.

48. Fuchigami A, Shigiyama F, Kitazawa T*, et al.* Efficacy of dapagliflozin versus sitagliptin on cardiometabolic risk factors in Japanese patients with type 2 diabetes: a prospective, randomized study (DIVERSITY-CVR). Cardiovasc Diabetol. 2020; **19**: 1.

49. Hayashi T, Fukui T, Nakanishi N*, et al.* Dapagliflozin decreases small dense low-density lipoprotein-cholesterol and increases high-density lipoprotein 2-cholesterol in patients with type 2 diabetes: comparison with sitagliptin. Cardiovasc Diabetol. 2017; **16**: 8.

50. Kayano H, Koba S, Hirano T*, et al.* Dapagliflozin influences ventricular hemodynamics and exercise-induced pulmonary hypertension in type 2 diabetes patients - A randomized controlled trial. Circ J. 2020; **84**: 1807-1817.

51. Nomoto H, Miyoshi H, Sugawara H*, et al.* A randomized controlled trial comparing the effects of dapagliflozin and DPP-4 inhibitors on glucose variability and metabolic parameters in patients with type 2 diabetes mellitus on insulin. Diabetol Metab Syndr. 2017; **9**: 54.

52. Phrommintikul A, Wongcharoen W, Kumfu S, Jaiwongkam T, Gunaparn S, Chattipakorn S. Effects of dapagliflozin vs vildagliptin on cardiometabolic parameters in diabetic patients with coronary artery disease: a randomised study. Br J Clin Pharmacol. 2019; **85**: 1337-1347.

53. Satirapoj B, Korkiatpitak P, Supasyndh O. Effect of sodium-glucose cotransporter 2 inhibitor on proximal tubular function and injury in patients with type 2 diabetes: a randomized controlled trial. Clin Kidney J. 2019; **12**: 326-332.

54. Shigiyama F, Kumashiro N. Effectiveness of dapagliflozin on vascular endothelial function and glycemic control in patients with early-stage type 2 diabetes mellitus: DEFENCE study. Cardiovasc Diabetol. 2017; **16**: 84.

55. Singh JSS, Mordi IR, Vickneson K*, et al.* Dapagliflozin versus placebo on left ventricular remodeling in patients with diabetes and heart failure: the REFORM trial. Diabetes Care. 2020; **43**: 1356-1359.

56. Lee SH, Min KW, Lee BW, Jeong IK, Yoo SJ. Effect of dapagliflozin as an add-on therapy to insulin on the glycemic variability in subjects with type 2 diabetes mellitus (DIVE): a multicenter, placebo-controlled, double-blind, randomized study. Diabetes Metab J. 2021; 45: 339-348.

57. Cho KY, Nakamura A, Omori K*, et al.* Effect of switching from pioglitazone to the sodium glucose co-transporter-2 inhibitor dapagliflozin on body weight and metabolism-related factors in patients with type 2 diabetes mellitus: An open-label, prospective, randomized, parallel-group comparison trial. Diabetes Obes Metab. 2019; **21**: 710-714.

58. Rosenstock J, Aggarwal N, Polidori D*, et al.* Dose-ranging effects of canagliflozin, a sodium-glucose cotransporter 2 inhibitor, as add-on to metformin in subjects with type 2 diabetes. Diabetes Care. 2012; **35**: 1232-1238.

59. Sha S, Polidori D, Heise T*, et al.* Effect of the sodium glucose co-transporter 2 inhibitor canagliflozin on plasma volume in patients with type 2 diabetes mellitus. Diabetes Obes Metab. 2014; **16**: 1087-1095.

60. Oshima M, Neuen BL, Jardine MJ*, et al.* Effects of canagliflozin on anaemia in patients with type 2 diabetes and chronic kidney disease: a post-hoc analysis from the CREDENCE trial. Lancet Diabetes Endocrinol. 2020; **8**: 903-914.

61. Inagaki N, Kondo K, Yoshinari T, Takahashi N, Susuta Y, Kuki H. Efficacy and safety of canagliflozin rnonotherapy in Japanese patients with type 2 diabetes inadequately controlled with diet and exercise: a 24-week, randomized, double-blind, placebo-controlled, Phase III study. Expert Opin Pharmacother. 2014; **15**: 1501-1515.

62. Inagaki N, Harashima S, Maruyama N, Kawaguchi Y, Goda M, Iijima H. Efficacy and safety of canagliflozin in combination with insulin: a double-blind, randomized, placebo-controlled study in Japanese patients with type 2 diabetes mellitus. Cardiovasc Diabetol. 2016; **15**: 89.

63. Takashima H, Yoshida Y, Nagura C*, et al.* Renoprotective effects of canagliflozin, a sodium glucose cotransporter 2 inhibitor, in type 2 diabetes patients with chronic kidney disease: A randomized open-label prospective trial. Diab Vasc Dis Res. 2018; **15**: 469-472.

64. Kashiwagi A, Kazuta K, Takinami Y, Yoshida S, Utsuno A, Nagase I. Ipragliflozin improves glycemic control in Japanese patients with type 2 diabetes mellitus: the BRIGHTEN study: BRIGHTEN: double-blind randomized study of ipragliflozin to show its efficacy as monotherapy in T2DM patients. Diabetol Int. 2014; **6**: 8-18.

65. Kashiwagi A, Kazuta K, Yoshida S, Nagase I. Randomized, placebo-controlled, double-blind glycemic control trial of novel sodium-dependent glucose cotransporter 2 inhibitor ipragliflozin in Japanese patients with type 2 diabetes mellitus. J Diabetes Investig. 2014; **5**: 382-391.

66. Kashiwagi A, Akiyama N, Shiga T*, et al.* Efficacy and safety of ipragliflozin as an add-on to a sulfonylurea in Japanese patients with inadequately controlled type 2 diabetes: results of the randomized, placebo-controlled, double-blind, phase III EMIT study. Diabetol Int. 2014; **6**: 125-138.

67. Kashiwagi A, Shiga T, Akiyama N*, et al.* Efficacy and safety of ipragliflozin as an add-on to pioglitazone in Japanese patients with inadequately controlled type 2 diabetes: a randomized, double-blind, placebo-controlled study (the SPOTLIGHT study). Diabetol Int. 2014; **6**: 104-116.

68. Kashiwagi A, Takahashi H, Ishikawa H*, et al.* A randomized, double-blind, placebo-controlled study on long-term efficacy and safety of ipragliflozin treatment in patients with type 2 diabetes mellitus and renal impairment: results of the long-term ASP1941 safety evaluation in patients with type 2 diabetes with renal impairment (LANTERN) study. Diabetes Obes Metab. 2015; **17**: 152-160.

69. Fonseca VA, Ferrannini E, Wilding JP*, et al.* Active- and placebo-controlled dose-finding study to assess the efficacy, safety, and tolerability of multiple doses of ipragliflozin in patients with type 2 diabetes mellitus. J Diabetes Complications. 2013; **27**: 268-273.

70. Wilding JP, Ferrannini E, Fonseca VA, Wilpshaar W, Dhanjal P, Houzer A. Efficacy and safety of ipragliflozin in patients with type 2 diabetes inadequately controlled on metformin: a dose-finding study. Diabetes Obes Metab. 2013; **15**: 403-409.

71. Lu CH, Min KW, Chuang LM, Kokubo S, Yoshida S, Cha BS. Efficacy, safety, and tolerability of ipragliflozin in Asian patients with type 2 diabetes mellitus and inadequate glycemic control with metformin: Results of a phase 3 randomized, placebo-controlled, double-blind, multicenter trial. J Diabetes Investig. 2016; **7**: 366-373.

72. Han KA, Chon S, Chung CH, Lim S. Efficacy and safety of ipragliflozin as an add-on therapy to sitagliptin and metformin in Korean patients with inadequately controlled type 2 diabetes mellitus: A randomized controlled trial. Diabetes Obes Metab. 2018; **20**: 2408-2415.

73. Kitazawa M, Katagiri T, Suzuki H*, et al.* A 52-week randomized controlled trial of ipragliflozin or sitagliptin in type 2 diabetes combined with metformin: the N-ISM study. Diabetes Obes Metab. 2021; 23: 811-821.

74. Seino Y, Sasaki T, Fukatsu A, Ubukata M, Sakai S, Samukawa Y. Dose-finding study of luseogliflozin in Japanese patients with type 2 diabetes mellitus: a 12-week, randomized, double-blind, placebo-controlled, phase II study. Curr Med Res Opin. 2014; **30**: 1231-1244.

75. Seino Y, Sasaki T, Fukatsu A, Sakai S, Samukawa Y. Efficacy and safety of luseogliflozin monotherapy in Japanese patients with type 2 diabetes mellitus: a 12-week, randomized, placebo-controlled, phase II study. Curr Med Res Opin. 2014; **30**: 1219-1230.

76. Seino Y, Sasaki T, Fukatsu A, Ubukata M, Sakai S, Samukawa Y. Efficacy and safety of luseogliflozin as monotherapy in Japanese patients with type 2 diabetes mellitus: a randomized, double-blind, placebo-controlled, phase 3 study. Curr Med Res Opin. 2014; **30**: 1245-1255.

77. Haneda M, Seino Y, Inagaki N*, et al.* Influence of renal function on the 52-week efficacy and safety of the sodium glucose cotransporter 2 inhibitor luseogliflozin in Japanese patients with type 2 diabetes mellitus. Clin Ther. 2016; **38**: 66-88.e20.

78. Seino Y, Sasaki T, Fukatsu A, Imazeki H, Ochiai H, Sakai S. Efficacy and safety of luseogliflozin added to insulin therapy in Japanese patients with type 2 diabetes: a multicenter, 52-week, clinical study with a 16-week, double-blind period and a 36-week, open-label period. Curr Med Res Opin. 2018; **34**: 981-994.

79. Gallo S, Charbonnel B, Goldman A. Long-term efficacy and safety of ertugliflozin in patients with type 2 diabetes mellitus inadequately controlled with metformin monotherapy: 104-week VERTIS MET trial. Diabetes Obes Metab. 2019; **21**: 1027-1036.

80. Terra SG, Focht K, Davies M. A Phase III, efficacy and safety study of ertugliflozin monotherapy in people with type 2 diabetes mellitus inadequately controlled with diet and exercise alone. Diabetes Obes Metab. 2017; **19**: 721-728.

81. Aronson R, Frias J. Long-term efficacy and safety of ertugliflozin monotherapy in patients with inadequately controlled T2DM despite diet and exercise: VERTIS MONO extension study. Diabetes Obes Metab. 2018; **20**: 1453-1460.

82. Ji L, Liu Y, Miao H*, et al.* Safety and efficacy of ertugliflozin in Asian patients with type 2 diabetes mellitus inadequately controlled with metformin monotherapy: VERTIS Asia. Diabetes Obes Metab. 2019; **21**: 1474-1482.

83. Halvorsen YC, Walford GA, Massaro J, Aftring RP, Freeman MW. A 96-week, multinational, randomized, double-blind, parallel-group, clinical trial evaluating the safety and effectiveness of bexagliflozin as a monotherapy for adults with type 2 diabetes. Diabetes Obes Metab. 2019; **21**: 2496-2504.

84. Halvorsen YD, Lock JP, Zhou W, Zhu F, Freeman MW. A 24-week, randomized, double-blind, active-controlled clinical trial comparing bexagliflozin with sitagliptin as an adjunct to metformin for the treatment of type 2 diabetes in adults. Diabetes Obes Metab. 2019; **21**: 2248-2256.
